# Tumor Microenvironment On‐A‐Chip and Single‐Cell Analysis Reveal Synergistic Stromal–Immune Crosstalk on Breast Cancer Progression

**DOI:** 10.1002/advs.202413457

**Published:** 2025-03-08

**Authors:** Kalpana Ravi, Yining Zhang, Lydia Sakala, Twinkle Jina Minette Manoharan, Barbara Pockaj, Joshua LaBaer, Jin G. Park, Mehdi Nikkhah

**Affiliations:** ^1^ School of Biological and Health Systems Engineering (SBHSE) Arizona State University Tempe AZ 85287 USA; ^2^ Biodesign Virginia G. Piper Center for Personalized Diagnostics Arizona State University Tempe AZ 85287 USA; ^3^ Department of Surgery Mayo Clinic Phoenix AZ 85054 USA

**Keywords:** cancer‐associated fibroblast (CAFs), macrophages, microfluidics, migration, scRNA sequencing, triple‐negative breast cancer (TNBC), tumor microenvironment (TME)

## Abstract

Solid tumors develop within a complex environment called the tumor microenvironment (TME), which is sculpted by the presence of other cells, such as cancer‐associated fibroblasts (CAFs) and immune cells like macrophages (Mφs). Despite the presence of immune cells, tumor cells orchestrate a tumor‐supportive environment through intricate interaction with the components of the TME. However, the specific mechanism by which this intercellular dialogue is regulated is not fully understood. To that end, the development of an organotypic 3D breast TME‐on‐a‐chip (TMEC) model, integrated with single‐cell RNA sequencing analysis, is reported to mechanistically evaluate the progression of triple‐negative breast cancer (TNBC) cells in the presence of patient‐derived CAFs and Mφs. Extensive functional assays, including invasion and morphometric characterization, reveal the synergistic influence of CAFs and Mφs on tumor cells. Furthermore, gene expression and pathway enrichment analyses identify the involvement of the *KYNU* gene, suggesting a potential immune evasion mechanism through the kynurenine pathway. Lastly, the pharmacological targeting of the identified pathway is investigated.

## Graphical abstract

Using a 3D tumor microenvironment (TME)‐on‐a‐chip model, tumor–stromal–immune crosstalk in triple‐negative breast cancer is studied, revealing the TME's combinatorial effect on cancer progression. Integrating single‐cell transcriptomic analysis demonstrates metabolic reprogramming via the kynurenine pathway, highlighting the complex TME cellular interactions that drive disease progression.

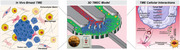

## 1 Introduction

Cancer is an immensely complex and heterogeneous disease that manifests within a dynamic multicellular ecosystem, allowing for sustained growth and survival.^[^ ^1^ ^]^ The tumor microenvironment (TME) is an intricate landscape composed of stromal cells, such as cancer‐associated fibroblasts (CAFs), and a rich diversity of immune cells, including tumor‐associated macrophages (TAMs), T lymphocytes, natural killer (NK) cells, as well as the extracellular matrix (ECM), along with cytokines, chemokines, and extracellular vesicles.^[^ ^2^ ^]^ The ever‐growing thrust to effectively treat cancer has led to the understanding of TME's contribution to cancer initiation and progression. For instance, CD8^+^ T cells, known for their strong anticancer capabilities, help create an immunosuppressive environment by immobilizing regulatory T cells.^[^ ^3^ ^]^ Similarly to normal organs, tumor cells secrete vascular endothelial growth factor A (VEGFA) to satisfy their nutrient and oxygen demands, resulting in the formation of new blood vessels through angiogenesis. As a result, endothelial cells show increased levels of inhibitory molecules that contribute to immunosuppression.^[^ ^4^ ^]^ These insights on TME capabilities in orchestrating tumor growth have unequivocally shattered the common treatment paradox, which focuses on neoplastic cancer cells.^[^ ^5^ ^]^ From the inception of TME research, the clear conceptualization has been to develop TME‐targeted therapies as a strategic approach to surmount the challenges posed by cancer. Initially, there was an unwavering optimism that targeting stromal and immune cells, which are genetically stable, would significantly reduce acquired resistance, a common issue with traditional treatment approaches.^[^ ^6^ ^]^ Furthermore, it was strongly believed that these therapies could universally benefit from a one‐size‐fits‐all concept.^[^ ^7^ ^]^

However, with the recent findings, it has become apparent that early assumptions were oversimplified, and targeting TME components is not a straightforward approach.^[^ ^8^ ^]^ For instance, tumors in distinct organs have tissue‐specific microenvironments.^[^ ^9^ ^]^ In the case of ovarian cancer, tumor lesions are highly infiltrated with T cells but correlate with a poor response to immunotherapies, suggesting a potential influence of other cells within the TME on the shielding of the treatment outcome.^[^ ^10^ ^]^ In the case of pancreatic ductal carcinoma, the depletion of CAFs from the TME has shown increased infiltration of regulatory T cells, ultimately resulting in immunosuppression.^[^ ^11^ ^]^ Similarly, therapeutic targeting of C─C chemokine ligand 2 (CCL2) in breast cancer (BC) using neutralizing antibodies has shown paradoxical effects, by increasing monocyte release and cancer cell metastasis.^[^ ^12^ ^]^ These examples inevitably provide evidence for the crucial influence of complex TME on disease progression, highlighting the critical effects of cellular crosstalk on treatment outcomes. Despite the promise of targeting TME, the utility of such a logical framework still faces notable roadblocks, mainly due to the diminished repertoire of refined model systems that allow for the recapitulation of the intricate cellular interactions between tumor, stromal, and immune cells within the TME niche.^[^ ^13^ ^]^

Specifically in the context of BC, the TME is regulated with high infiltration of two key cellular components the CAFs and macrophages (Mφs).^[^ ^13a^ ^]^ CAFs are the central component of the tumor stroma, profoundly involved in various stages of cancer progression through active interaction with the tumor cells.^[^ ^14^ ^]^ CAFs are highly plastic cells that perform myriad functions, from synthesis to remodeling of ECM, altering the behavior of cancer cells and have strong immunomodulatory functions.^[^ ^15^ ^]^ These complex and intertwined behaviors of CAFs make them a critical player in cancer progression.^[^ ^16^ ^]^ Next to CAFs, Mφs are the most potent immune cells found within the breast TME that are known to influence cancer mechanics, including the promotion of cancer cell proliferation, invasion, and altering the innate and adaptive immune responses.^[^ ^17^ ^]^ Mφs are components of innate immunity, that are co‐opted by the tumor cells to create an environment conducive to their growth.^[^ ^18^ ^]^ For decades, Mφs were considered phagocytic cells with antitumor capabilities.^[^ ^17^ ^]^ However, recent studies have led to the apparent findings that Mφs are functionally distinct cells that are highly responsive to the environmental cues present within the TME. The phenotype of Mφs is fostered by the growing tumor and accordingly, Mφs could be classified as antitumor cells that release pro‐inflammatory cytokines (M1) and/or protumor cells that release anti‐inflammatory cytokines (M2) phenotypes, representing the extremities of polarized state.^[^ ^19^ ^]^

Given this wealth of information, various approaches have been taken to therapeutically target and deplete the key breast TME components, namely CAFs and Mφs. For example, inhibitors of colony‐stimulating factor 1 receptor (CSF1R) are proposed as an alternative to reeducate Mφs with tumoricidal properties.^[^ ^5a^ ^]^ In that line, CAFs are targeted through surface marker expression for direct depletion within the TME.^[^ ^5a^ ^]^ Frustratingly, these approaches have exhibited subpar efficacy in treating cancer due to their failure to account for the synergistic influence of cellular crosstalk. Furthermore, their low efficacy in clinical outcomes can be attributed to the use of 2D‐based and in‐vivo‐based models. While 2D‐based assays have contributed to critical cancer discoveries, these models are oversimplified and neglect the influence of the local environment in which cancer thrives.^[^ ^20^ ^]^ On the other hand, in vivo models such as mice xenografts offer system‐level complexities but fall short of genetic variations impacting outcomes.^[^ ^21^ ^]^ To conquer these challenges, it is imperative to employ a multidisciplinary and high‐resolution approach that encompasses advanced model systems and technologies to comprehend the complexities of cancer with increasing precision.

In this context, microfluidic‐based tumor‐on‐chip (TOC) models have offered unprecedented potential to closely mimic the TME in a well‐controlled manner, allowing delineation of the complex tumorigenesis process.^[^ ^13^, ^22^ ^]^ For example, our previous study by Truong et al. investigated the interplay of CAFs in the realm of BC and reported the molecular and functional changes in the cancer cell as a result of tumor–stromal crosstalk.^[^ ^23^ ^]^ Another study investigated the communication between Mφs and mesothelial cells using a TOC platform. This study revealed that upregulation of inflammatory protein‐1β expression in Mφs causes mesothelial cells to express P‐selectin, suggesting a potential molecule to inhibit metastasis.^[^ ^24^ ^]^ Along the same line, a biomimetic hydrogel‐based parallel microfluidic model was devised to assess the influence of Mφs on breast cancer invasion. Findings from this study highlighted the involvement of paracrine and juxtacrine signaling in cancer and Mφ crosstalk.^[^ ^25^ ^]^ Though these examples highlight the significant contribution of microfluidic models to cancer research, there remains a critical need for a more advanced and refined in vitro model that allows recapitulation of the complex environmental cues induced by the cooperation of CAFs and Mφs on tumor progression.

In this work, we describe the development of a 3D organotypic breast tumor microenvironment‐on‐a‐chip (TMEC) model integrated with single‐resolution studies to mechanistically decipher the tripartite crosstalk of CAF, Mφ, and triple‐negative breast cancer (TNBC) cells in driving tumor progression. To tease out the individual and collective influence of stromal, immune, and tumor cells, we built our model with well‐controlled and increasing experimental complexity of the cell type. The established TMEC model system was assessed for various functional analyses such as tumor invasion, real‐time migration, and cellular morphometric changes. We further mechanistically elucidated the tumor–stromal–immune crosstalk using single‐cell RNA sequencing (scRNA‐seq). Through scRNA analysis, we validated the clinical relevance of our model system and further identified key ligand–receptors (L–R) and differentially expressed genes (DEGs), which underscore tumor progression, as a consequence of cellular communication. Together, this study demonstrates the recapitulation of dynamic in vivo cellular interactions within the TME and the practical applicability of our organotypic model system in identifying the drivers of tumor progression at single‐cell resolution, unveiling discoveries that have not been elucidated in the past.

## 2 Results

### 2.1 Characterization of Patient‐Derived CAFs and Mφs

CAFs were isolated from triple‐negative breast cancer tissue samples, as described in our previous study.^[^ ^23^ ^]^ Studies were conducted after receiving proper approval from the Institutional Review Board (IRB) at Mayo Clinic of Arizona. To ensure proper maintenance of CAFs’ property in vitro, cells were used within passage 10 and were routinely screened for fibroblast marker expressions, namely alpha‐smooth muscle actin (𝛂‐SMA) and cytokeratin. Immunofluorescent (IF) characterization of CAFs showed positive expression for 𝛂‐SMA and negative expression of cytokeratin (Figure S1A, Supporting Information). Thus, these marker expressions by CAFs confirmed their maintenance of the cellular property throughout their culture period.

To derive naïve Mφs, THP‐1 monocytes were differentiated by incubating cells with 50 ng mL^−1^ of phorbol 12‐myristate‐13‐acetate (PMA) containing Roswell Park Memorial Institute Medium (RPMI) for 24 h, followed by 24 h of rest phase in RPMI media without PMA. Post differentiation, adherent cells were stained for the cluster of differentiation 68 (CD68) markers through IF staining. Positive expression of CD68 by adherent cells confirmed the successful differentiation of monocytes to Mφs (Figure S1B, Supporting Information). These routine screenings of stromal and immune cells ensured proper differentiation and maintenance of cellular components before incorporation into the TMEC model.

### 2.2 Recapitulation of Breast TME Interactions with a Triculture TMEC Model

**Figure 1:**
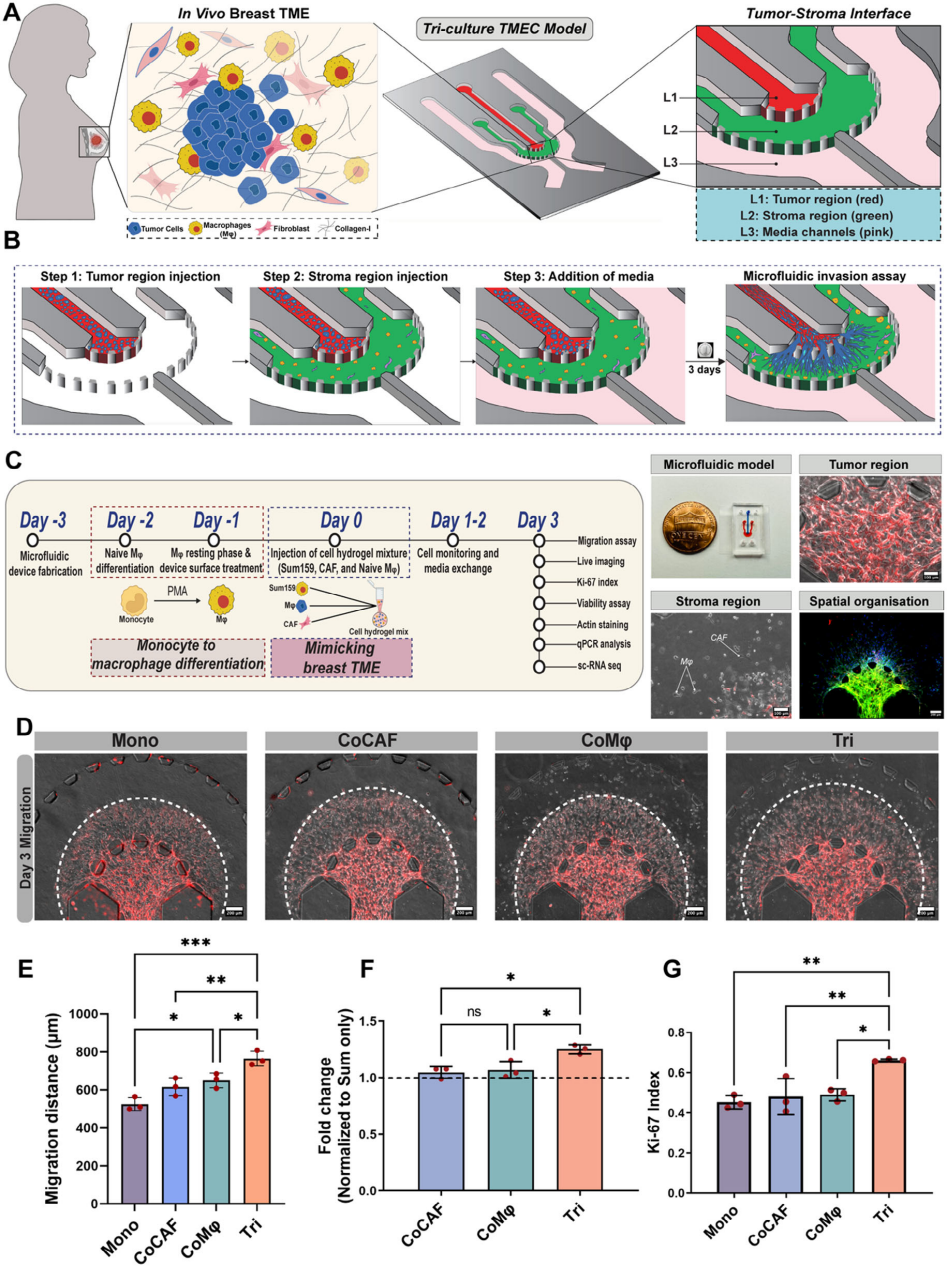
Breast TME‐on‐a‐chip (TMEC) for studying cellular crosstalk in cancer progression. A) Schematic representation of in vivo breast TME with essential stromal components (left) (created with BioRender.com), an overview of the 3D microfluidic breast TME‐on‐a‐chip model consisting of three channels: tumor region (red), stroma region (green), and flanking media channels. B) Graphical representation of establishment of 3D TMEC triculture invasion assay. C) Experimental workflow detailing the timeframe for macrophage differentiation, reconstruction of breast TME, and other downstream analysis (left). Photograph of the microfluidic device next to a cent to depict the size of the model, an overlaid image of m‐Cherry expression Sum159 cells (TNBC cells) within the tumor region representing the dense tumor, and distribution of CAFs and Mφs in the stroma region of the device, and F‐actin (green) stained image of the TMEC model depicting the interconnectivity between tumor and stroma region (right). D) Phase‐contrast images showing the migratory potential of cancer cells in the presence and absence of CAFs and Mφs, day 3 (after interaction with other cells). White dashed line represent the maximum migration distance of the cancer cells. E) Quantification of migration distance of Sum159 cells under different culture conditions. *p < 0.05, **p < 0.01, One‐way ANOVA with Tukey's multiple comparisons test, *n =* 3, biological replicates per condition. F) Quantitative analysis of normalized fold change of Sum159 migration distance (normalized to sum (Mono condition)). *p < 0.05. One‐way ANOVA with Tukey's multiple comparisons test, *n =* 3, biological replicates. G) Quantification of proliferative index (Ki‐67 index) of Sum159 cells under specified conditions. *p < 0.05, **p < 0.01, one‐way ANOVA with Tukey's multiple comparisons test, *n* = 3 biological replicates. ANOVA, analysis of variance.

We primarily chose CAFs and Mφs as the stromal components for this study for two key reasons. First, CAFs and Mφs are the most abundant cellular populations within the breast TME.^[^ ^26^ ^]^ Second, the reciprocal communication between CAFs and Mφs aids in the establishment of an immunosuppressive milieu.^[^ ^26a^ ^]^ Though a clear perspective exists on the collaborative roles of CAFs and Mφs in driving tumor growth, the synergistic effect of tumor cells, CAFs, and Mφs has not been studied at the single‐cell level using well‐controlled ex vivo model systems. Therefore, to mechanistically understand the contributions of CAFs and Mφs in driving tumors, we utilized an organotypic TMEC model that allows the incorporation of TNBC cells, CAFs, and Mφs (**Figure** **1**A).

The proposed organotypic TMEC model enabled the spatial organization of a tumor region surrounded by a stroma region, allowing continuous interaction of tumor–stromal cells, closely mimicking the in vivo heterotypic cellular interactions. The triculture (Tri) microfluidic invasion assay was established by arranging the breast cancer cells into the central tumor region (red) and incorporating the stromal cells (CAFs and Mφs) into the surrounding stroma region (green), where both regions contained 3D ECM (Figure 1A). Specifically, the tumor region was loaded with Sum159 cells embedded in a hydrogel mixture containing collagen I and Matrigel at a ratio of 1:1 (Figure 1B). Collagen and Matrigel were chosen due to their close resemblance to breast ECM composition. Subsequently, the stromal region was formed based on different experimental conditions, namely, i) monoculture (Mono), ii) CoCAF, iii) CoMφ, and iv) Tri (Table S1, Supporting Information). These well‐controlled experimental conditions enable sequentially increasing the model complexity in a defined manner to gain a deeper understanding of the cellular crosstalk within the TME (Table S1, Supporting Information).

### 2.3 Assessment of Dynamic Role of TME Interaction on Cancer Invasion

Upon establishing the triculture invasion model, we primarily investigated the influence of CAFs and Mφs on the invasion and aggressiveness of cancer cells. In all conditions, Sum159 cells were allowed to interact with the stromal components for 3 days, and the migration of cancer cells into the stroma region was observed by phase‐contrast imaging (Figure 1C; Figure S2, Supporting Information). Tumor cells invaded the stroma within 24 h of injection under all conditions. However, the presence of stromal cells significantly influenced the migratory potential of cancer cells compared to the monoculture condition (Figure 1D). Tumor cells exhibited increased migration in both CoCAF and CoMφ conditions, with a statistically significant increase in CoMφ condition compared to monoculture. Notably, the highest migration of tumor cells was observed in the Tri condition compared to all other conditions (Figure 1E). Analysis of normalized migration distance demonstrated a significantly increased invasiveness of the cancer cells in the Tri condition, compared to both CoCAF and CoMφ conditions, further confirming the synergistic influence of CAFs and Mφ on cancer cell invasion (Figure 1F).

Several studies have reported that the presence of CAFs and Mφs in the TME is associated with high proliferation of cancer cells, suggesting a supportive role of these cellular components in fueling tumor growth.^[^ ^27^ ^]^ Therefore, we next interrogated the proliferative potential of cancer cells within our model system using Ki‐67 staining in response to stromal components. Analysis of the Ki‐67 index across different conditions showed increased expression in the Tri condition compared to all the other conditions (Figure 1G). Taken together, these findings indicate the synergistic influence of CAFs and Mφs on cancer cell invasion and proliferation underscoring the crucial role of TME components and crosstalk on tumor progression.

### 2.4 Real‐Time Migration of Tumor and Stromal Cells in 3D

**Figure 2:**
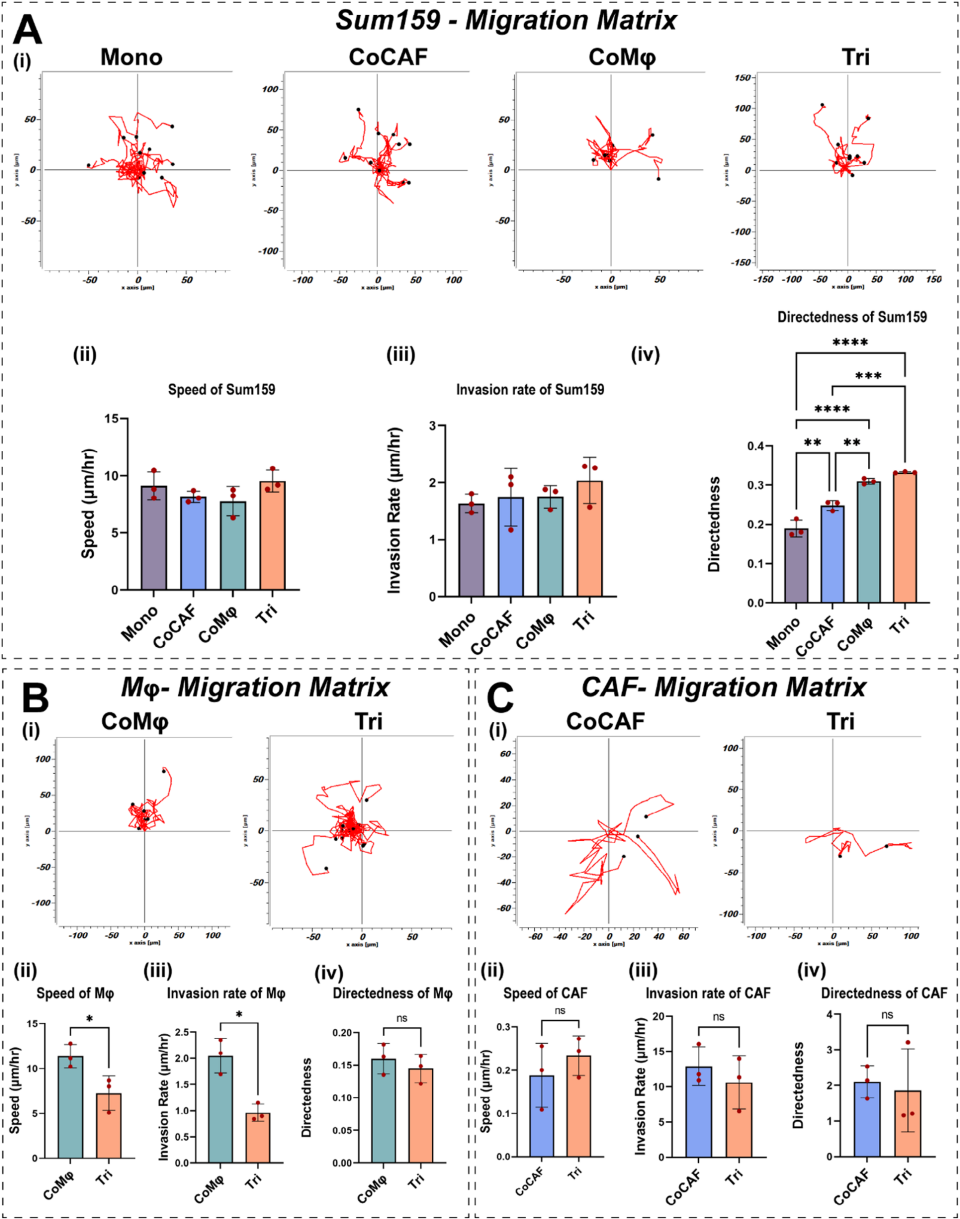
Quantification of real‐time migration matrix of cancer, CAFs, and Mφs. A) Migration behavior of Sum159 cells: i) trajectory plots of cancer cells across different conditions; ii–iv) quantification of speed, invasion rate, and directedness of cancer cells. B) Migration potential of Mφs: i) migration tracks of Mφs from CoMφ and Tri; ii–iv) quantification of speed, invasion rate, and directedness of Mφs in the presence of tumor and CAFs. C) Migration potential of CAFs: i) migration tracks of CAFs from CoCAF and Tri; ii–iv) quantification of speed, invasion rate, and directedness of CAFs in the presence of tumor and Mφs. **p < 0.01, ***p < 0.001, ****p < 0.0001. One‐way ANOVA with Tukey's multiple comparisons test, *n* = 3 biological replicates.

Leveraging the optical transparency of our TMEC model, we monitored the motility and migration of each cell type within the 3D tissue at single‐cell resolution. All live images were taken on days 2–3 of migration and individual cells were traced using the ImageJ plugin followed by migration matrix quantification using Ibidi chemotaxis tool (**Figure** **2**A(i)). Analysis of the speed and invasion of tumor cells across conditions showed no statistical difference (Figure 2A(ii,iii); Movie S1, Supporting Information). Furthermore, we quantified other matrices of cancer cells such as directedness. Interestingly, tumor cells in the presence of stromal components (CoCAF, CoMφ, and Tri) showed significantly increased directedness as compared to monoculture (Figure 2A(iv); Movies S2,S4, and S6, Supporting Information). Notably, the presence of CAF and Mφ in Tri condition increased the directional migration dynamics of Sum159 cells, underscoring the complex motility of the tumor cells influenced by the co‐presence of stromal and immune cells (Figure 2A(iv); Movie S6, Supporting Information).

Next, we quantified similar migration matrices of immune (Mφs) and stromal cells (CAFs). The behavior of Mφ was assessed between CoMφ and Tri, whereas the behavior of CAFs was compared between CoCAF and Tri conditions. Evaluation of speed and invasion rate of Mφ across conditions displayed an enhanced migration speed and invasion rate in CoMφ condition compared to Tri (Figure 2B(i–iii); Movies S5 and S8, Supporting Information). However, we did not observe any significance in the Mφ directedness (Figure 2B(iv)). Analysis of CAF speed, directedness, and invasion rate showed no significant differences across conditions. Similar to Mφ, we observed a near significant (*p*‐value) increase in the speed and invasion rate of CAFs in CoCAF condition (Figure 2C(i–iv); Movies S3 and S7, Supporting Information). These findings corroborate well with our previous findings, where we observed increased speed of CAFs in the presence of tumor cells.^[^ ^23^ ^]^ Taken together, these analyses of tumor and stromal cell migration matrices highlight the intricate interplay of cellular components within the TME.

### 2.5 Morphological Characterization of Cancer Cells in the 3D TMEC Model

**Figure 3:**
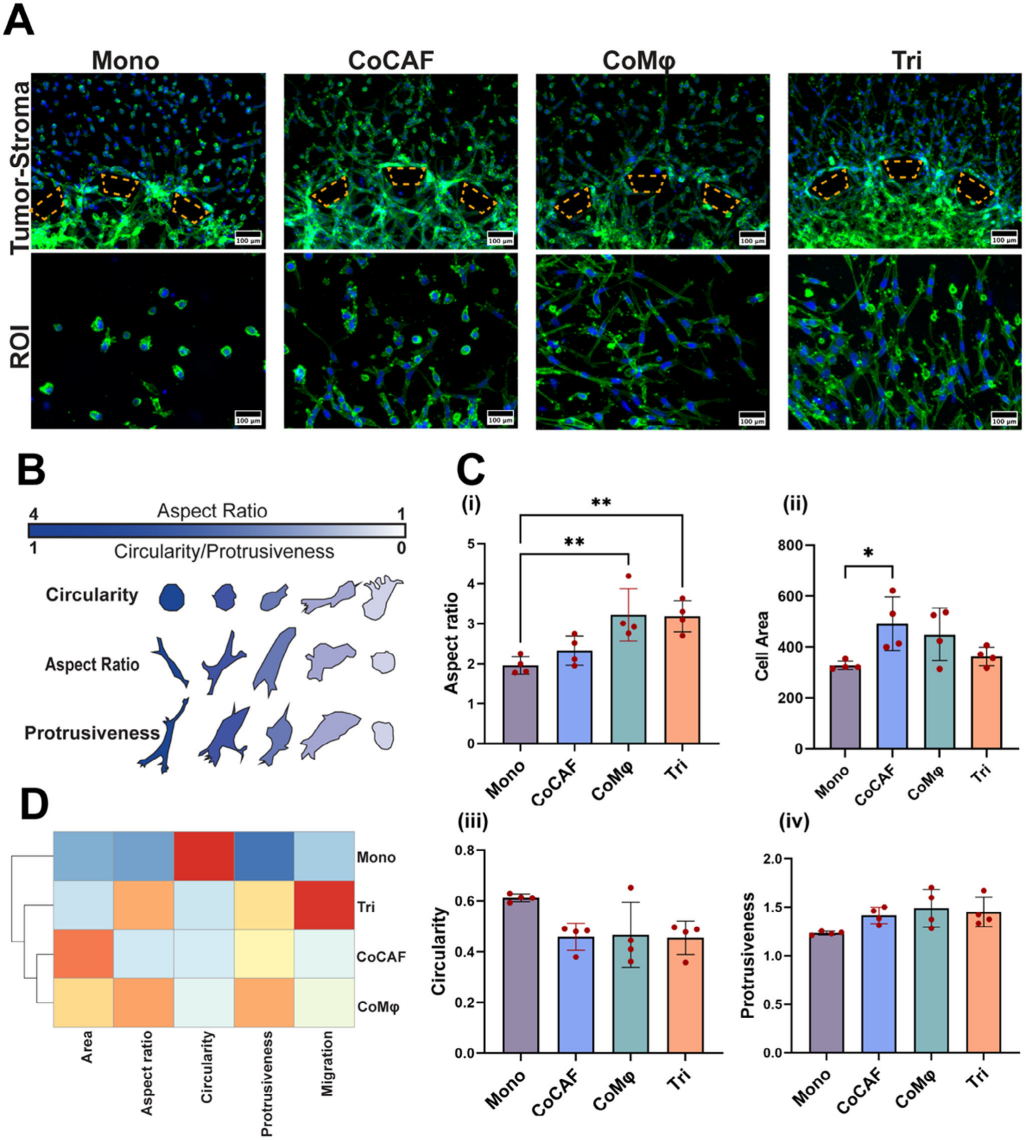
Morphological characterization of cytoskeletal organization of cancer cells. A) Top: Fluorescent images of actively invading cancer cells stained with F‐actin (green) and DAPI (blue). Bottom: Representative image showing the shape of cancer cells from each condition. B) Graphical representation of the shape description parameter. C) Quantification of morphological parameters: i) aspect ratio, ii) cell area, iii) circularity, and iv) protrusiveness of cancer cell shape. D) Unsupervised clustering heat map of shape descriptor and migration distance of cancer cells from different culture conditions. *p < 0.05, **p < 0.01. One‐way ANOVA with Tukey's multiple comparisons test, *n* = 4 biological replicates.

Cancer cells migrating into the stroma displayed a distinct morphology, which was observed by real‐time migration analysis (**Figure** **3**A). To further tease the influence of stromal cells on the phenotypic signature of cancer cells, we analyzed their morphological changes through F‐actin staining. Various morphological parameters such as aspect ratio (AR), cell area (area), circularity, and protrusiveness were quantified to derive meaningful conclusions (Figure 3B).

Under different conditions, the tumor cells exhibited distinct morphologies. Specifically, cells within Mono condition were observed to be more rounded with increased circularity and reduced AR, whereas cells in CoCAF exhibited a mixed population of round and elongated cells as evidenced by increased area and protrusiveness, similar to our previous study.^[^ ^23^ ^]^ Contrary to these groups, tumor cells in the presence of Mφs adapted an elongated morphology with increased AR, protrusiveness, and reduced area, and circularity (Figure 3C(i–iv)).

Next, we performed an unsupervised hierarchical clustering analysis of both migration distance and morphological parameters (Figure 3D). Clustering analysis revealed a distinct pattern. First, co‐culture conditions (CoCAF and CoMφ) clustered together, highlighting the individual impact of each stromal and immune cell. Conversely, the Tri condition clustered separately, underscoring the synergistic influence of CAFs and Mφs on the morphology and migratory behavior of cancer cells. Mono condition clustered distinctly apart from all other groups, emphasizing the lack of cellular interactions. These findings highlight the impact of immune and stromal cells of the TME on the morphological and migratory dynamics of cancer cells.

### 2.6 Single‐Cell Transcriptomics Analysis of the Breast TMEC Model

**Figure 4:**
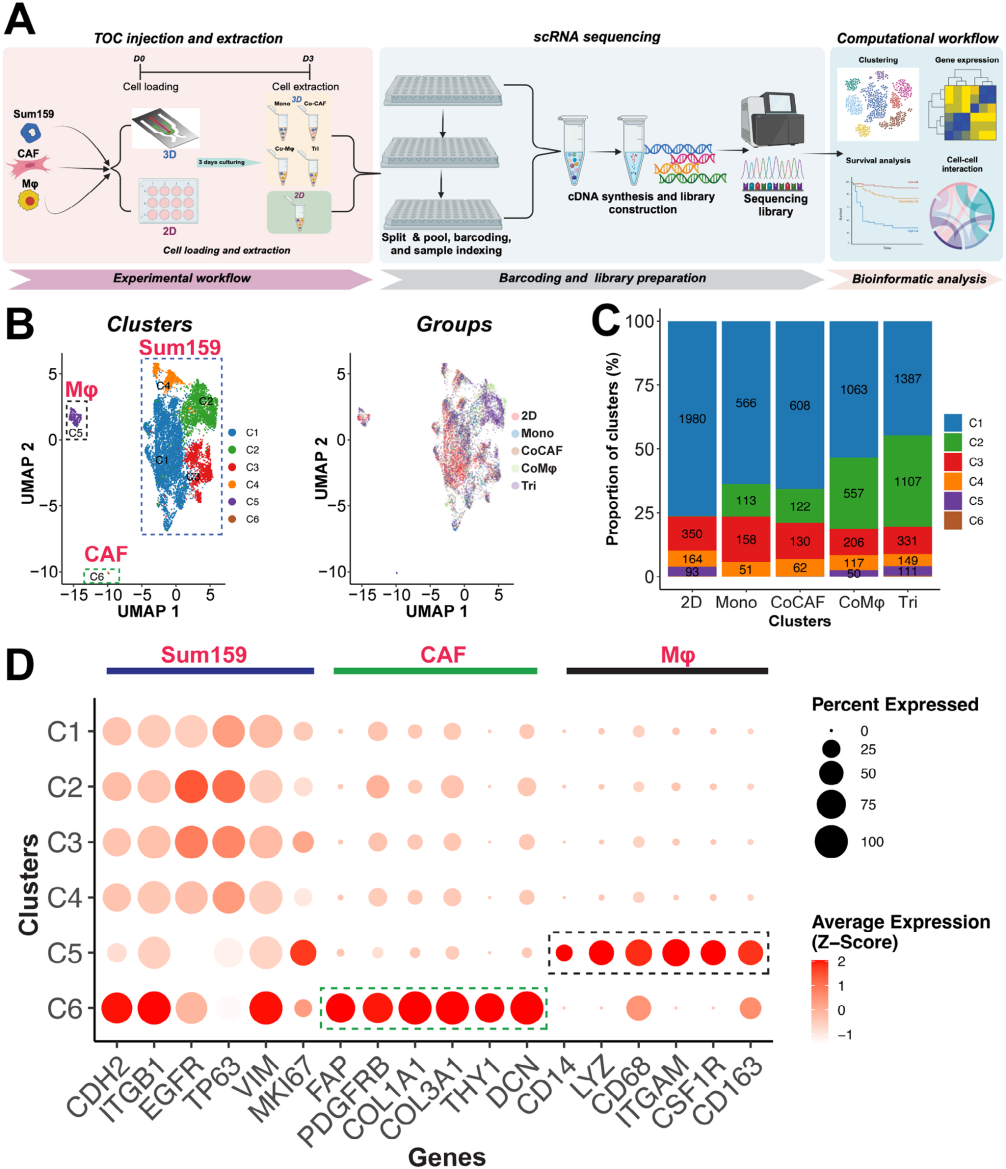
Single‐cell profiling of 3D TMEC model. A) Schematic overview of the experimental workflow for scRNA‐sequencing with details of cell extraction, barcoding, and library preparation (created with BioRender.com). B) UMAP visualization of cells from the 2D culture and 3D TMEC model annotated for cell clusters (left) and samples (right). C) Bar plot illustrating the distribution of cell proportion across clusters for each sample. D) Dot plot of canonical marker genes of cancer cells, CAFs, and Mφs for annotation of the cell cluster.

Functional assessments, including migration and morphometric analysis, confirmed the intricate interplay of immune, stromal, and tumor cells and their impact on cancer cell aggressiveness. Besides this, gene expression analysis showcased the diverse phenotype of Mφs as a result of interaction with cancer cells and CAFs. Intrigued by these observations, we integrated our TMEC model system with scRNA‐seq analysis to mechanistically and systemically investigate the molecular dynamics regulating cellular communications. scRNA‐seq was performed on samples extracted from the TMEC model upon 3 days of invasion, alongside samples cultured in standard tissue culture plastic (2D) (**Figure** **4**A). RNA libraries were constructed, sequenced, and further downstream analysis was carried out.

We profiled a total of 9773 cells from both 3D and 2D samples, which included 6903 cells from the 3D TMEC model and 2597 cells from the 2D samples, following our quality control criteria. Potential doublets within the samples were removed prior to any analysis. After adjusting for cell cycle and batch effects, we performed unsupervised clustering and canonical marker gene expression to identify specific cell types in the sequenced samples. Using graph‐based clustering, we identified a total of six distinct cell clusters as shown in the uniform manifold approximation and projection (UMAP) plot (Figure 4B). Additionally, when the distribution of various cell types under different culture conditions across the identified clusters was examined (Figure 4C; Figure S3A, Supporting Information), cells from both 2D and 3D samples (Mono, CoCAF, CoMφ, and Tri) were distributed differently across the clusters (Figure S3B, Supporting Information). Specifically, cells from the 2D sample were predominantly found in C1, whereas the 3D cells were more broadly distributed to other clusters (Figure S3A,B, Supporting Information).

Next, to identify and distinguish different cell types within the clusters, we performed a canonical marker gene expression analysis. Through this analysis, we identified correctly three key cell types expressing the marker genes within our sequenced samples, Sum159 (*EGFR*, *VIM*, *CDH2*, *MKI67*, *ITGB1*, and *TP63*), CAF (*FAP*, *PDGFRB*, *COL1A1*, *THY1*, *DCN*, and *COL3A1*), and Mφ (*CD14*, *CD68*, *ITGAM*, *LYZ*, *CSF1R*, and *CD13*) (Figure S3C, Supporting Information). Further, examination of the marker expression in each cluster led to the annotation of C5 as Mφ, C6 as CAF, and the remaining clusters C1–C4 as Sum159 cells (Figure 4D; Figure S3C, Supporting Information). Notably, we observed four distinct clusters of the tumor cells, underscoring the heterogeneous population of these cells. The cell proportion analysis showed that C5 (Mφ) and C6 (CAF) were represented by 2D samples as well as 3D TMEC CoCAF, CoMφ, and Tri samples. Importantly, *CDH2* and *ITGB1*, which are implicated in influencing tumor progression, were found to be enriched in the CAF cluster, which is consistent with previous studies.^[^ ^28^ ^]^ Taken together, these findings validated the successful capture and sequencing of all cell types from the 2D and the 3D TMEC model, accurately reflecting their respective culture conditions.

### 2.7 Transcriptomic and Pathway Analysis of Cancer Cells in the 3D TMEC Model and 2D Culture

**Figure 5:**
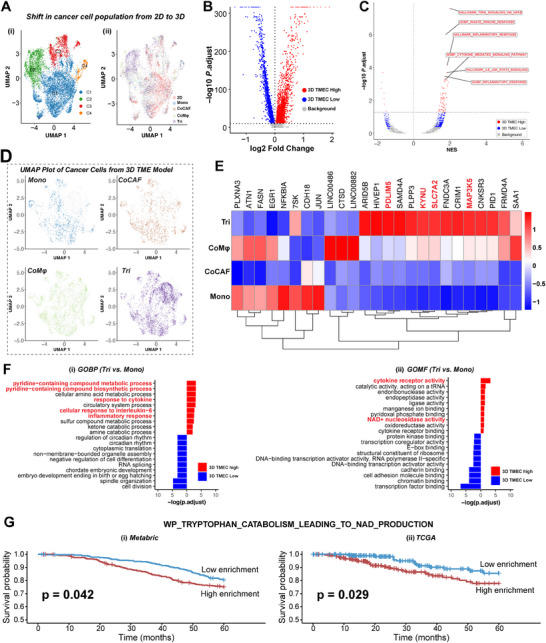
Transcriptomic profiling of cancer cells. A) UMAP plot showing the distribution for i) clusters and ii) samples. B) Volcano plot of DEGs between cancer cells in 3D versus 2D. *p*.adjust values in the Y‐axis were obtained from *t‐*tests. Red: upregulated genes in 3D (*p*.adjust < 1e^−10^), blue: downregulated genes in 3D (*p*.adjust < 1e^−10^). C) Volcano plot of pathway enrichment levels in DEGs between cancer cells in 3D and 2D. NES from GSEA on each cell (*X*‐axis) and the adjusted *p*‐values from *t*‐test (*Y*‐axis) are shown. Red: *p*.adjust < 0.05, blue: *p* < 0.05. D) UMAP plot of cancer cells showing the distribution of cancer cells from each culture condition. Each colored dot represents the distribution of the cells in clusters. E) Heat map of top 25 DEGs across four different culture conditions in 3D. Color represents *Z*‐score transformed log2 expression level. F) Gene Ontology (GO) analysis of i) Biological Processes (BP) and ii) Molecular Functions (MF) of cancer cells from Tri condition compared to Mono condition. Red: enriched top ten pathways in upregulated genes in 3D, blue: enriched top ten pathways in downregulated genes in 3D. G) Kaplan–Meier analysis of overall survival of TNBC patients with high (top 20%) or low (bottom 20%) enrichment of the tryptophan catabolism pathways in i) Metabric and ii) TCGA databases.

To validate the effectiveness of our TMEC model system in studying molecular mechanisms of cell–cell crosstalk at the single‐cell level and recapitulating cancer manifestations in vivo, we performed a comparative transcriptomic analysis of cancer cells from the 3D TMEC model alongside 2D samples. Re‐clustering of only cancer cells in both 3D and 2D conditions identified four clusters (C1–C4) (**Figure** **5**A(i)). Among those, C1 predominantly contained cells from the 2D samples, while the other clusters (C2–C4) were composed of cells from the 3D TMEC model (Figure 5A(ii); Figure S4A,B, Supporting Information). To gain insights into the gene expression profile differences between cancer cells in 3D versus 2D, we next performed a differential gene expression analysis. To ensure the robustness of our analysis, we employed both parametric and nonparametric statistical tests: the *t*‐test and the Wilcoxon rank‐sum test. This analysis identified a total of 4857 DEGs from the *t‐*test (Figure 5B) and 4954 DEGS from the Wilcoxon test (Figure S4C, Supporting Information). Even with strict thresholding and the adjusted *p*‐value of <1e^−10^, 2103 and 2754 genes were up‐ and downregulated, respectively (shown in red and blue, respectively, in Figure 5B) and 1510 and 3444 genes were up‐ and downregulated, respectively (shown in red and blue, respectively, in Figure S4C of the Supporting Information) in the 3D samples, indicating a stark difference in global gene expression profiles of cancer cell phenotype in our 3D TMEC model compared to standard 2D samples. Specifically, DEGs in 3D showed high expression of genes involved in nuclear factor kappa‐B (NF‐κB) signaling (*TNFAIP3*, *NFKB1*, *RELB*, and *TMEPAI*),^[^ ^29^ ^]^ epithelial‐mesenchymal transition (EMT) (*S100A4*),^[^ ^30^ ^]^ immune function‐related genes (*NNMT*, *CSF‐1*, and *CXCL2*),^[^ ^31^ ^]^ and integrin‐mediated genes (*ITGB8*)^[^ ^32^ ^]^ (Tables S2,S3, Supporting Information). To further probe into changes in biological processes and pathways, we performed gene set enrichment analysis (GSEA) and observed that the DEGs were intricately linked to cancer‐related pathways such as cytokine‐mediated signaling, inflammatory response, tumor necrosis factor‐alpha (TNF‐α) signaling via NF‐κB, interferon‐gamma response,^[^ ^33^ ^]^ and interleukin‐6 (IL‐6) Janus kinases signal transducer and activator of transcription (IL‐6 JAK‐STAT) pathway^[^ ^33b^ ^]^ (Figure 5C; Figure S4D and Tables S4 and S5, Supporting Information). Additionally, to gain more insights into the dominant biological processes at play among the cancer cells, we performed a pseudo‐bulk analysis by effectively aggregating cells within each sample and conducting the same pathway enrichment analysis (Figure S4E, Supporting Information). Consistently, findings from the pseudo‐bulk analysis decisively revealed the enrichment of TNF‐α signaling, cytokine‐mediated signaling pathways, and inflammatory responses within the 3D TMEC samples (Figure S4E and Table S6, Supporting Information). These findings suggest a significant involvement of key pathways related to chronic inflammation and tumor progression, confirming the enrichment of cancer‐associated processes within the TMEC 3D environment.^[^ ^34^ ^]^ Notably, cancer hallmark pathways were more enriched in cancer cells from the 3D TMEC model compared to standard 2D cultures (Figure 5C; Figure S4D,E, Supporting Information). Furthermore, GSEA on the Gene Ontology (GO) Biological Process (GOBP) terms decisively highlighted key biological processes such as “vasculature development,” “epithelium migration,” “toll‐like receptor signaling,” “cellular response to cytokine,” “and ‘innate immune response” were enriched in the 3D samples (Figure S4F and Table S7, Supporting Information).

### 2.8 Functional Influence of CAFs and Mφs on Cancer Cells in the 3D TMEC Model

To further understand the dynamic influence of CAFs and Mφs on tumor progression and the underlying molecular mechanisms, we next determined the changes in gene expression profiles of cancer cells from the 3D TMEC model in different culture conditions (Figure 5D–F; Figure S4G, Supporting Information). Clustering analysis of cells from the 3D TMEC model based on gene expression values identified three specific clusters of cancer cells (Figure S4G(i,ii), Supporting Information). To better visualize the distribution of cells from each condition, we further plotted the cell density in the UMAP space (Figure 5D), which effectively illustrated and summarized the difference in cancer cell gene expression profiles across Mono, CoCAF, CoMφ, and Tri conditions. Furthermore, by comparing one condition with others through multiple pairwise comparisons, we identified a total of 1136 DEGs from the *t*‐test and 1146 DEGs from the Wilcoxon rank‐sum test (Table S8, Supporting Information). The heat map of the top 25 DEGs showed a distinct expression pattern across different conditions, suggesting the unique influence of stromal cells on cancer cell phenotype (Figure 5E; Figure S4H, Supporting Information). Notably, genes such as kynureninase (*KYNU*), PDZ, and LIM domain protein *(PDLIM5)*, mitogen‐activated protein kinase kinase kinase 5 (*MAP3K5*), and solute carrier family 7 member 2 (*SLC7A2*) were upregulated in Tri condition compared to all other conditions (Figure 5E; Figure S4H, Supporting Information), suggesting a combinatorial influence of CAFs and Mφs. Particularly, the *KYNU* gene has been reported to be involved in the biosynthesis of nicotinamide adenine dinucleotide (NAD) co‐factors from tryptophan (Trp) through the kynurenine pathway (KP); has been strongly implicated in inflammation; and is a key player in mediating immune evasion.^[^ ^35^ ^]^ Complementing to this, several studies have reported that *KYNU* is related to immune system diseases,^[^ ^36^ ^]^ central nervous system disease,^[^ ^37^ ^]^ and various types of cancer.^[^ ^38^ ^]^ Furthermore, *SLC7A2* has been reported to be involved in tumor survival and adaptations to the TME, particularly.^[^ ^39^ ^]^ These compelling findings underscore the combinatorial influence of CAFs and Mφs on the molecular signatures of cancer, driving it toward an aggressive phenotype.

To further gain mechanistic insights into the differential pathway enrichment profiles among the various 3D TMEC samples (Mono, CoCAF, CoMφ, and Tri), we performed the single‐sample GSEA (ssGSEA) to further explore the altered biological pathways and processes. Notably, in the synergistic presence of CAFs and Mφs (i.e., Tri condition), differentially enriched pathways involved “tryptophan catabolism leading to NAD production,” “kynurenine pathway,” “regulation of toll‐like receptor signaling pathway,” “positive regulation of macrophage activation,” and “regulation of reactive oxygen species” (Figure S5A,B and Table S9, Supporting Information). The enrichment of immune response indicates the dynamic interplay of cancer cells with the stromal cells. Importantly, the enrichment of tryptophan catabolism and kynurenine‐related pathways indicates the metabolic reprogramming of the tumor cells to sustain complex TME interactions. Moreover, KYNU is a key enzyme in the kynurenine pathway of tryptophan catabolism, and its upregulation may drive increased immunosuppressive metabolite production, contributing to tumor progression. Taken together, these findings emphasize the combinatorial influence of stromal cells on the molecular alterations of cancer cells. Furthermore, the use of two statistical analyses reinforces the robustness and reliability of our findings.

To further dissect the synergistic influence of stromal CAF and Mφ in driving tumor progression, we also performed a GSEA analysis for GOBP and GO Molecular Function (GOMF) on cancer cells, particularly from Mono and Tri conditions (Figure 5F). Notably, our findings identified the involvement of key biological processes such as response to “IL‐6,” “pyridine‐containing compound metabolic process,” and “cytokine receptor binding” in Tri condition, demonstrating the response of cancer cells to a plethora of signaling cues from the surrounding stromal component (Figure 5F(i); Tables S10,S11, Supporting Information). It is well known that CAFs are the major source of IL‐6 within the TME, where it has been implicated in contributing to tumor growth and metastasis.^[^ ^40^ ^]^ Furthermore, the presence of IL‐6 in TME can polarize Mφ toward the M2 phenotype, implying the synergistic influence of tumor–stromal–immune crosstalk in aiding tumor progression.^[^ ^33b^ ^]^ Another hallmark capability of cancer is to switch the energy metabolism. Intriguingly, our data demonstrate the enrichment of some well‐known metabolic processes such as the “NAD^+^ activity,” “pyridine‐containing compound biosynthetic process,” as well as “pyridine‐containing compound metabolic process.” NAD has been shown to play a pivotal role in aiding the metabolic switch of cancer cells from oxidative phosphorylation to glycolysis.^[^ ^41^ ^]^ Additionally, KP is the primary route of NAD^+^ biosynthesis from tryptophan, and the enrichment for pyridine biosynthesis suggests an elevated flux through this pathway, possibly due to tumor‐driven metabolic rewiring^[^ ^42^ ^]^ (Figure 5F(ii); Tables S10,S11, Supporting Information). In conclusion, GOBP and GOMF analyses revealed nuanced mechanisms by which cancer cells interact with stromal components within our TMEC model, further advancing our understanding of tumor biology in the context of TME.

Finally, to further corroborate the clinical relevance of the tryptophan and NAD pathways identified from the TMEC, we compared the pathway enrichment with clinical outcome of TNBC patients using publicly available clinical datasets, Cancer Genome Atlas (TCGA) and Metabric. Specifically, we performed ssGSEA analysis on individual TNBC tumor samples and comparative survival analysis using the top and the bottom 20% of samples based on the normalized enrichment scores (NES). As shown in Figure 5G, high expression of tryptophan catabolism genes in the “tryptophan catabolism leading to NAD production” pathway was associated significantly with poor overall survival with *p*‐values of 0.042 and 0.029, for Metabric and TCGA, respectively, indicating that this pathway contributes to more aggressive tumor behavior and poor prognosis (Figure 5G(i,ii)). These results from patient survival analysis and our transcriptomic profiling validate the relevance of the 3D TME model in capturing clinically significant molecular features of cancer.

### 2.9 Delineation of Macrophage Phenotype in 3D TMEC

**Figure 6:**
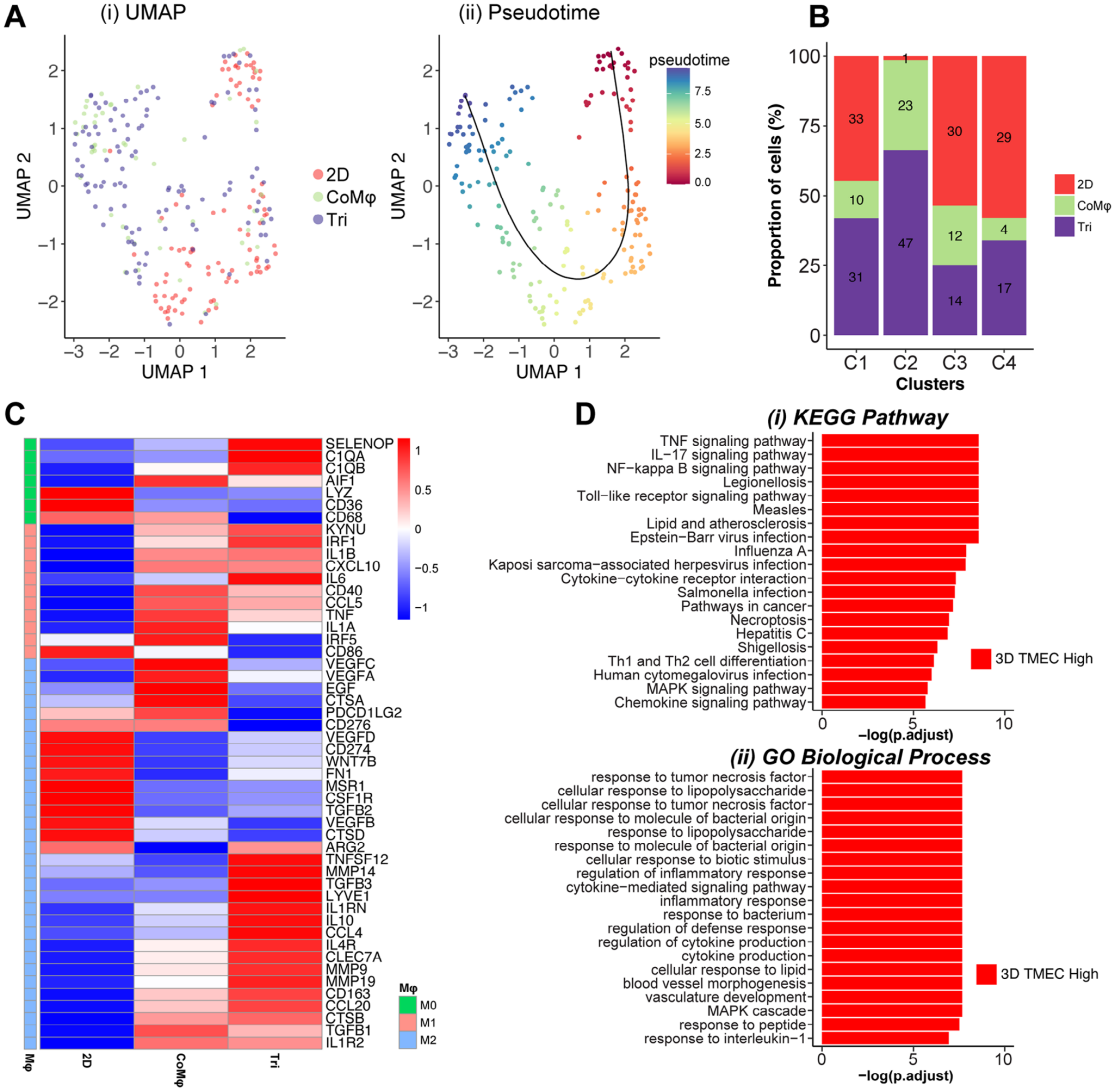
Trajectory analysis of the polarized state of macrophages. A) Mφ trajectory inference: i) UMAP of Mφs from 3D and 2D samples; ii) pseudotime trajectory analysis of macrophages done by Slingshot. The red dot represents the initial starting point, and the blue dot represents the end of the trajectory. B) Bar plot illustrating the distribution of macrophages across each cluster. C) Heat map of signature M0, M1, and M2 marker gene expressions of macrophages. Color represents *Z*‐score transformed log2 expression level. D) Bar plot of the top terms in i) KEGG and ii) GOBP databases that are enriched in upregulated (red) or downregulated (blue) DEGs in macrophages in 3D compared to 2D. The *p.adjust* values are from GSEA.

Mφs play a central role in tumorigenesis, and within the TME, these cells exhibit in two antagonistic states namely the pro‐inflammatory M1 and anti‐inflammatory M2. To that end, we next investigated how the crosstalk within the TME influences Mφ phenotype. To better understand the fate of Mφ within the TMEC model, we next focused on the phenotypic signature of Mφs from both the 3D TMEC model (CoMφ and Tri) and 2D samples, using 169 known macrophage marker genes for clustering and UMAP analysis, from which 4 clusters (C1–C4) of Mφs were identified (**Figure** **6**A; Figure S6A and Tables S12,S13, Supporting Information), with cluster C2 containing cells predominantly from the 3D samples, cluster 4 (C4) with 2D samples, and the remaining clusters (C1 and C3) contributed by the cells from both 3D TMEC model and 2D samples (Figure 6B; Figure S6B, Supporting Information).

To gain insights into the functional states of Mφs, we performed the trajectory inference on the Mφs UMAP clusters (Figure 6A(ii); Figure S6C, Supporting Information). A pseudotime trajectory analysis (using *Slingshot* and *Monocle3*) was then performed to visualize the transition states of Mφs in each condition, by using C4 (dominated by 2D cells) as the initial timepoint to assess their progression over psuedotime. This revealed that Mφs undergo continuous phenotypic and transcriptional reprogramming as they transition from a 2D to a 3D environment, following a single trajectory (Figure 6A(ii); Figure S6C, Supporting Information). Importantly, the trajectory led to a terminal cluster predominantly composed of macrophages from 3D TMEC (C2), reiterating the phenotypic transition of these cells within the 3D TME.

Furthermore, we conducted cluster‐based pathway enrichment analysis (Figure S6D, Supporting Information). In the early stage (C4) compared to other clusters, we observed enrichment for “T‐cell activation,” “pathways in cancer,” and “focal adhesion,” suggesting a primed but less physiologically relevant state (Figure S6D, Supporting Information). In contrast, at the later trajectory point (3D Mφs–C2), pathways such as “toll‐like receptor,” “cytokine interaction,” “NF‐κB signaling pathway,” and “TNF‐α signaling” became enriched, indicating a shift toward an immune‐responsive and activated state (Figure S6D and Table S14, Supporting Information). This underscores the 3D TMEC in modulating a functional Mφ state through tumor–stromal interaction and leading to immune responses that influence tumor progression or suppression. Additionally, to gain insights into transcriptional changes occurring during the transitional state, we performed a GSEA along the pseudotime trajectory (Figure S7, Supporting Information). Specifically, we reclustered the samples using the four highly correlated (positive and negative) genes (Figure S7A,B and Table S15, Supporting Information). This pseudotime trajectory analysis coupled with GSEA highlighted NF‐κB as the major signaling pathway driving the Mφ transition (Figure S7C and Table S15, Supporting Information). Previous studies have reported the crucial role of NF‐κB in Mφ polarization in cancer.^[^ ^43^, ^44^ ^]^ Our finding further demonstrates that Mφ within the 3D TMEC model becomes increasingly activated, exhibiting a pro‐inflammatory phenotype, contributing to tumorigenesis.

We also performed a sample‐based DEG analysis for Mφs on the 3D TMEC models (CoMφ and Tri) and 2D cultures using pairwise comparisons to profile the polarized state of the Mφs. Overview of the DEG heat map highlighted the differences across the 2D, CoMφ, and Tri conditions, emphasizing the unique pattern of Mφs in 3D versus 2D (Figure S8A, Supporting Information). Concomitantly, Mφs in CoMφ and Tri exhibited a similar expression pattern and clustered together, whereas 2D samples formed a separate cluster. Additionally, to determine the activation state of the Mφs across conditions, we examined the relative expression of canonical M0, M1, and M2 signatures and other recently identified macrophage subtype markers^[^ ^45^ ^]^ such as apolipoprotein (*APOE)*, secreted phosphoprotein 1(*SPP1)*, C‐X‐C motif chemokine ligand 10 (*CXCL10)*, and interleukin‐4 receptor *IL4R* (Figure 6C; Figure S8B and Tables S12,S13, Supporting Information). The heat map of marker signature expression revealed distinct expression patterns across the conditions (Figure 6C; Figure S8B, Supporting Information). Particularly, the expression of the pan‐macrophage marker (CD68) was relatively high in 2D Mφ compared to 3D Mφ (Figure 6C). We also observed a baseline expression of certain M1 and M2 marker genes in 2D Mφs. This is consistent with previous findings, showing specific marker gene expression by Mφs upon stimulation of monocytes to naïve Mφs.^[^ ^46^ ^]^ Conversely, Mφs from the 3D TMEC model showed expression of several M1 and M2 marker genes, and specific gene signatures like complement 1q A (*C1QA)*, *SPP1*, selenoprotein P (*SELENOP)*, tubulin alpha‐1b (*TUBA1B)*, aquaprin‐9 (*AQP9)*, and thrombospondin‐1 (*THBS1)* indicating the differentiation of naïve Mφ, with unique expression patterns based on the environmental conditions.

Our scRNA analysis consistently showed higher expression of classic M2 marker expression in Tri condition (Figure 6C). Activated M2 Mφs typically expresses high levels of CD163, and matrix metalloproteinases (MMP) like MMP9 and MMP19, thereby aiding in cell adhesion and promoting tumor growth.^[^ ^47^ ^]^ Importantly, we also observed the upregulation of subtype‐specific genes such as *SPP1*, *C1QA*, *C1QB*, *C1QC*, *AQP9*, and *THBS1* in Tri condition compared to CoMφ (Figure S8B, Supporting Information). SPP1‐expressing macrophages have been associated with the M2‐like phenotype in the context of breast cancer, which has been implicated in promoting cancer cell metastasis and growth. This indicates the presence of subtype‐specific Mφ population within our TMEC model (Figure S8B, Supporting Information).^[^ ^48^ ^]^

Furthermore, a distinct subtype, namely complement 1*q* (C1Q^+^) Mφs with high expression of C1QA, C1QB, and C1QC, has recently been identified in various cancers playing crucial roles in modulating an immunosuppressive TME by regulating T‐cell function.^[^ ^49^ ^]^ Consistent with these studies, we observed a higher expression of C1Q^+^ Mφ’s signatures, including *C1QA*, *C1QB*, and *C1QC*, within our Tri samples. Additionally, all these subtype‐specific Mφs co‐expressed *CD163*, interleukin‐10 (*IL10)*, and *MMP*, underscoring their pro‐tumorigenic phenotype, similar to previous studies.^[^ ^50^ ^]^ It is also worth noting that Mφs in our model expressed pro‐inflammatory markers (M1) such as *CXCL10*, *IL4R*, and *CXCL8* indicative of hybrid activation with both pro‐inflammatory and pro‐tumorigenic characteristics (Figure 6C; Figure S8C, Supporting Information). Furthermore, several other marker genes that are known to support tumor growth and creating an immune suppressive environment like *IL6*, *IL10*, *C1QA*, interleukin‐1 receptor antagonist (*IL1RN)*, and *KYNU* were highly expressed in our Tri culture condition. Similarly, a comparison of cytokine gene expression between 3D versus 2D samples revealed similarities between cytokine profiles in 3D conditions (CoMφ and Tri) as opposed to the 2D samples (Figure S8C, Supporting Information). Specifically, cytokines involved in tumor progression such as *CXCL8*, *EBI3*, and *IL1RN* were upregulated in the triculture samples compared to other conditions (Figure S8D, Supporting Information). These findings imply that CAFs and tumor cells collaborate to skew the Mφs to exacerbate an environment that is conducive to tumor growth rather than supporting immune‐mediated destruction.^[^ ^51^ ^]^

Next, to validate the findings from scRNA‐seq on macrophage polarized state, using quantitative polymerase chain reaction (qPCR), we performed confirmatory gene expression analysis of the 3D samples from the TMEC model collected upon 72 h of interaction (Figure S9A, Supporting Information). As a control, Mφ alone (Mono (Mφ)) was cultured within the same 3D TMEC model without the presence of other cell types (Table S16, Supporting Information). We screened for an array of M1‐specific genes, namely IL6, TNF‐α, and inducible nitric oxide synthase (iNOS), as well as M2‐specific genes, including CD163, CD206, and IL10, along with the pan‐macrophage marker CD68 (Table S17, Supporting Information). Our qPCR analysis revealed a significant upregulation of CD163 expression by Mφs in Tri condition compared to those in CoMφ and Mφ alone (Figure S9B, Supporting Information). Furthermore, the Mφs also exhibited notable expression of other genes associated with the M1 phenotype, specifically TNF‐α, indicating a shift toward a pro‐inflammatory state (Figure S9B, Supporting Information). Overall, the comparison of relative gene expression across different conditions revealed higher expression of both M1 and M2 markers in Tri as well as CoMφ conditions (Figure S9C, Supporting Information). This observation on mixed M1/M2 population of Mφs could be attributed to the multifaceted interactions of these cells with surrounding CAFs and cancer cells, confirming the findings from scRNA analyses of Mφs.

To further identify the key biological pathways affected by culture conditions, we performed a functional enrichment analysis of the DEGs between 2D and 3D Mφs (i.e., Tri and CoMφ). DEGs upregulated in 3D samples were predominantly enriched in immune‐associated and cancer‐associated terms, such as “IL‐17,” “NF‐κ B signaling,” “TNF signaling,” “mitogen‐activated protein kinase (MAPK) signaling,” “cytokine–cytokine interaction,” “pathways in cancer,” and “vascular development” (Figure 6D(i,ii); Tables S18,S19, Supporting Information). Together, the results from analysis on Mφs reveals the crosstalk between Mφs and tumor cells within our 3D TME model indicating the critical role of Mφs in promoting tumor progression.^[^ ^52^ ^]^

### 2.10 Characterization of Cell–Cell Crosstalk between Cancer Cells, CAFs, and Macrophages

**Figure 7:**
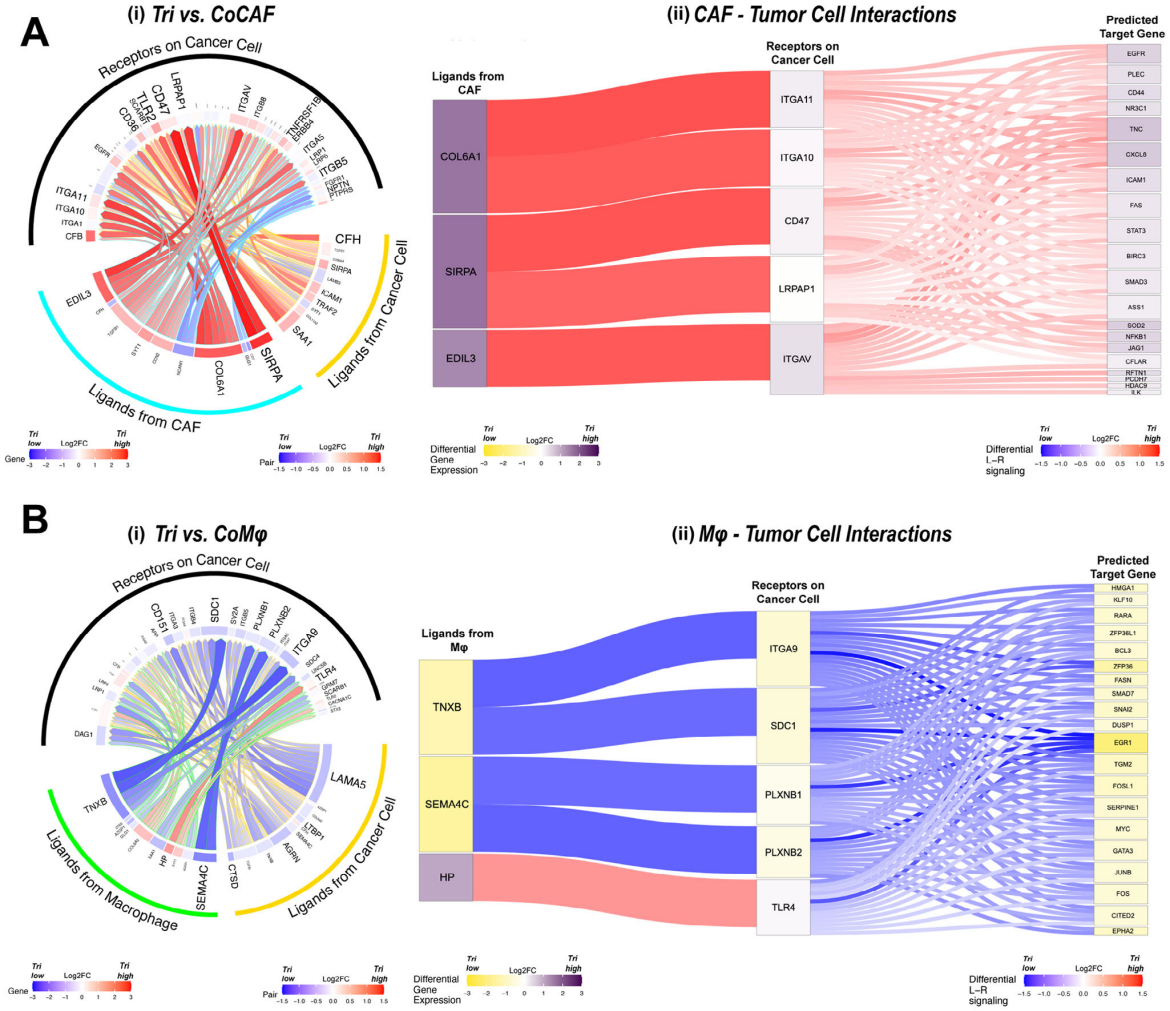
Cell–cell interaction of CAFs, macrophages, and tumor cells within 3D TMEC. A) Differential L–R interaction between Tri and CoCAF conditions induced by addition of Mφs: i) Circos plot of top ten differential CAF‐to‐cancer (cyan ring) and autocrine (yellow ring) L–R signaling. ii) Sankey plot shows differential expression of the top CAF‐to‐cancer L–R pairs and the predicted target genes in cancer cells. B) Differential L–R interaction between Tri and CoMφ conditions induced by addition of CAF: i) Circos plot of top differential Mφ‐to‐cancer (green ring) and autocrine (yellow ring) L–R signaling; ii) Sankey plot shows differential expression of the top Mφ‐to‐cancer L–R pairs and the predicted target genes in cancer cells.

To investigate how the presence of both stromal CAFs and Mφs and their interaction influence tumor cells, we further performed a ligand–receptor (L–R) pair analysis. L–R analysis between Tri versus CoMφ condition as well as Tri versus CoCAF conditions was conducted using “NicheNet.”^[^ ^53^ ^]^ Specifically, by comparing Tri versus CoCAF, our objective is to evaluate the alterations in L–R interactions between CAFs and tumor cells driven by the presence of Mφs. Comparison between Tri versus CoCAF led to the identification of 75 L–R pairs from CAFs and 84 pairs from tumor cells corresponding to top 20 ligands with highest activity. Next, we selected the top 10 ligands for both CAF–tumor and tumor–tumor interactions from the top 20 most active ligands, considering their receptor in each pair and visualized them using a Circos plot (**Figure** **7**A(i); Figure S10, Supporting Information). Additionally, to ensure robustness in our findings, we compared the L–R pairs identified by an alternative tool “CellChat.”^[^ ^54^ ^]^ Both NicheNet and CellChat can construct and analyze cell–cell communication networks based on L–R interactions. However, NicheNet also considers gene regulatory networks and the relationship between L–R interactions and downstream target genes, allowing it to predict downstream gene regulation. CellChat applies a stricter criterion for selecting differential pairs, resulting in a smaller set of L–R pairs, all of which were also consistently identified by NicheNet (Table S20, Supporting Information), confirming the robustness of our analysis (Figure S10, Supporting Information).

One of the top ligands induced by Mφs (i.e., expressed higher in CAFs in the Tri condition than CoCAF) was signal regulatory protein alpha (SIRPA), which is a well‐known ligand that mediates cancer immune evasion by activating the CD47 expression on cancer cells. The SIRPA–CD47 axis protects cancer cells from phagocytosis.^[^ ^55^ ^]^ Though this L–R interaction is well known, little has been investigated about the expression of SIRPA ligands on CAFs. Another Mφ‐induced ligand was EGF‐like repeats and discoidin domains 3 (EDIL3). Expression of EDIL3 by CAFs has been reported to support immune evasion in melanoma.^[^ ^56^ ^]^ EDIL3 binds strongly to the integrin receptor (ITGAV) on tumor cells. Integrin‐based cell surface receptors play a key role in promoting cancer cell invasion and migration through ECM remodeling, and overexpression of ITGAV has been reported in breast cancer as well.^[^ ^57^ ^]^ We speculated that activation of the EDIL3–ITGAV axis could potentially evade immune cell function and promote migration of cancer cells through integrin‐based ECM remodeling. Similarly, the collagen type V1 alpha 1 chain (COL6A1)–ITGA10, COL6A1–ITGA11, and transforming growth factor beta 1 (TGFB1)–TGFBR3 axis of L–R interaction were upregulated, suggesting a potential interaction with the ECM and an influence on immune cell phenotype. Furthermore, a comparison of L–R pairs utilizing CellChat revealed an overlap of 107 common L–R pairs. Particularly several L–R pairs involved in integrin‐mediated receptors were identified, reinforcing the robustness of the biological finding (Figure S10A(i) and Table S20, Supporting Information).

To further explore the downstream targets of these cellular interactions, we also examined expression of the predicted targets of identified L–R pairs (Figure 7A(ii)). Sankey plot of top L–R interactions between Tri and CoCAF conditions identified tenascin C (TNC) and CXCL8 to be the specific target genes downstream. Intriguingly, TNC is an ECM glycoprotein that is involved in tissue remodeling and is often upregulated in TME.^[^ ^58^ ^]^ On the other hand, CXCL8 is the gene that encodes for IL‐8, which has been reported to be associated with TNBC brain metastasis and immune infiltration.^[^ ^59^ ^]^

We conducted a similar analysis comparing Tri and CoMφ conditions to identify L–R pairs between cancer cells and Mφs that were altered by CAFs (Figure 7B(i)). In particular, Mφs demonstrated higher expression of the haptoglobin (HP) complex in Tri condition, which is highly expressed in M2‐type macrophages. The HP ligand strongly interacts with toll‐like receptor‐4 (TLR‐4). Activating TLR‐4 on cancer cells can increase the secretion of IL‐6 and enhance invasion abilities.^[^ ^60^ ^]^ In addition, it has been firmly established that TLR‐4 expression regulates integrin expression, effectively aiding in adhesion and invasion.^[^ ^61^ ^]^ Additionally, L–R pair analysis revealed 111 common pairs between Nichenet and CellChat (Figure S10 and Table S20, Supporting Information). Particularly, L–R pair identified through CellChat revealed the activation of THBS1–CD47 axis (Figure S10A(ii), Supporting Information). Recently, THBS1 expression by macrophages has been reported to promote tumor progression.^[^ ^62^ ^]^ Overall, the analysis for L–R interaction altered by CAFs has unequivocally identified multiple receptors on cancer cells that actively facilitate ECM degradation, immune evasion, and promote migration and invasion. Notably, converged upregulation of CD47 via the SIRPA–CD47 axis in CAF–tumor interaction (identified through NicheNet) as well as the THBS1–CD47 axis between Mφs and tumor cells in the presence of CAFs (identified from CellChat) indicates that upregulation of CD47 signaling is the main effect of stromal cells in TME, which may lead to immune evasion. Furthermore, as shown in a Sankey plot (Figure 7B(ii)), comparison of Tri versus CoMφ identified two highly downregulated downstream genes in cancer cells, early growth response 1 (EGR1) and zinc finger protein (ZFP36), which have been reported to be downregulated in patients with TNBC.^[^ ^63^ ^]^ These findings further demonstrate the clinical relevance of our 3D TMEC model.

### 2.11 Metabolic Flux Analysis

Pathway enrichment analysis of 3D cancer cells revealed the significant metabolic reprogramming within our TMEC model, specifically through the activation of *KYNU* gene and the promotion of tryptophan catabolism. To further validate the metabolic dysregulation contributing to cancer progression, we applied a computational metabolic flux analysis (MFA), using the METAFlux,^[^ ^64^ ^]^ our scRNA‐seq data. Comparative analysis of metabolic activity on the cancer cells at the pathway level across different culture conditions revealed significantly higher metabolic activity in Tri samples compared to other groups. Notably, pathways relevant to “fatty acid oxidation,” “fatty acid activation,” “trypthophan biosynthesis,” and “tryptophan metabolism” were enriched in Tri samples (Figure S11A, Supporting Information). To gain deeper understanding into the influence of the Trp pathway and the role of *KYNU* gene, we utilized the human metabolic atlas to identify the specific pathways impacted by *KYNU*. This identified the involvement of KYNU in the tryptophan biosynthesis subsystem namely HMR04224, HMR04220, and HMR04225.

**Figure 8:**
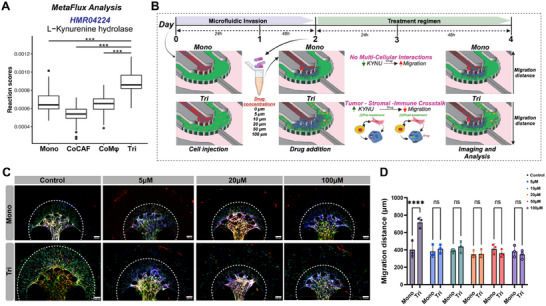
scRNA‐seq validation of identified metabolic pathway through small molecule drug inhibition. A) MetaFlux analysis shows the predicted flux scores of *KYNU*‐regulated reaction (HMR04224) in each sample. * *p*.adjust < 1e^−2^, ** *p*.adjust < 1e^−6^, *** *p*.adjust < 1e^−10^, ns *p*.adjust > 1e^−2^. B) Schematic representation of the experimental workflow for the model establishment for testing Ro‐61‐8048, detailing experimental conditions, treatment regimen highlighting the drug concentrations, and duration of the experiment focusing on cellular interaction as well as imaging time points (created with BioRender.com). C) Images of the TMEC model post‐treatment under various drug dosages (green: actin; red: Sum159 cancer cells, blue: DAPI nuclei), image captured on day 4. D) Comparison of cancer cell invasion quantified by migration distance between different culture conditions. ****p < 0.001, two‐way ANOVA with Tukey's multiple comparisons test, *n =* 3, biological replicates per condition.

Next, MFA was performed to estimate the flux of each metabolic reaction in the *KYNU*‐related pathways. For HMR04224, which represents the conversion of kynurenine to alanine and anthranilate, a positive reaction score was observed in the Tri samples, suggesting the active contribution of *KYNU* in kynurenine (**Figure** **8**A). Similarly, the flux scores from the other two reactions were consistent, further supporting the functional impact of stromal cells on the tryptophan catabolism within our model (Figure S11B,C, Supporting Information). These findings imply that KYNU may play a significant role in tumor progression and immune evasion, highlighting its potential as a therapeutic target.

### 2.12 Small Molecule Drug Validation on Inhibition of Tumor Invasion

To test the hypothesis that the enzymes in the KP/Trp pathway would be a potential target to inhibit tumor invasion, we then tested a small‐molecule drug that would block the Trp/KP pathway within our tumor on‐a‐chip model (Figure 8A). Specifically, after treating the cells in Mono and Tri conditions with different dosages of Ro‐61‐8048 that targets the upstream enzyme of the KP/Trp pathway (Figure 8B), we monitored the invasion of cancer cells into the stromal region for 48 h post‐treatment and compared the migration distance relative to the control (no drug) group as well as between conditions. Post drug treatment, a clear difference in the migration distance across conditions was observed (Figure 8B; Figure S12A, Supporting Information). Specifically, cancer cells in the drug‐treated Mono condition showed no difference in migration compared to the control condition (Figure S12B, Supporting Information), whereas cancer cells in the drug‐treated Tri condition showed a significant reduction in migration distance as compared to the control condition (Figure S12B, Supporting Information). Next, to represent the effect of the treatment regime on the migratory behavior of cancer cells, we also compared the migration distance between Mono and Tri for each drug concentration (Figure 8C). This analysis further confirmed that cancer cells in Tri condition migrated significantly higher compared to the Mono condition. On the contrary, upon inhibition of the KP/Trp pathway, we observed a reduction in the migration distance of the cancer cell in Tri to an extent similar to the Mono condition (Figure 8D).

Expanding on this, we also investigated the influence of Ro‐61‐8048 on each of stromal and immune cells through viability analysis. To evaluate this impact, we added the drug within our model across all the four culture conditions. Particularly, we tested three drug concentrations, which revealed no impact on the viability of the CAFs and Mφs (Figure S12C, Supporting Information). Importantly, this analysis also revealed that the drug impacted the cancer cell migration and did not actively involve in killing the tumor cells. This assay clearly validated our findings from our tumor on‐a‐chip studies and scRNA‐seq analyses, where *KYNU* is responding to drive cancer cell migration in the presence of stromal and immune cells through activation of the KP pathway.

## 3 Discussion

The environment in which the cancer cells thrive synergistically influences the growth of tumors as well as treatment outcomes.^[^ ^65^ ^]^ With the advancement of research, strategies for treating cancer have shifted toward addressing cancer as a whole, including its supporting niche. Despite continuous strides taken to eradicate cancer, it is ever‐evolving, ultimately leading to treatment failure. Among various reasons, cellular regulation and crosstalk within the TME have been attributed to sculpting a microenvironment favorable for tumor growth and poor response to treatment.^[^ ^66^ ^]^ Therefore, it is crucial to mechanistically investigate the influence of cellular communication within TME to improve treatment efficacy.

With the rise of advanced interdisciplinary technologies, models like microfluidic TMEC systems are quickly garnering attention in the field of cancer biology due to their accurate recapitulation of complex TME features.^[^ ^13^, ^67^ ^]^ Additionally, such models overcome limitations of interspecies variability and ethical concerns that are commonly associated with animal‐based models.^[^ ^68^ ^]^ Such state‐of‐the‐art microfluidic systems that allow spatial control, superior physiological relevance, and integration with downstream molecular assays hold promise to study the mechanism of TME interactions that contribute to immune evasion and cancer progression in the context of breast cancer.^[^ ^68^ ^]^

Leveraging the principles of microscale technologies, our research team has developed a unique TOC model that enables spatial organization of tumor region surrounded by stroma region.^[^ ^23^, ^69^ ^]^ This architecture offers the advantage of greater physiological relevance and captures the intricate cellular crosstalk of the TME. The devised model has been extensively validated for its applicability in the field of cancer bioengineering.^[^ ^23^, ^70^ ^]^ Notably, utilizing a similar two‐layered TOC model, our group demonstrated the effectiveness of our system in mimicking patient‐specific TME to evaluate the molecular events in tumor–stromal interactions.^[^ ^23^ ^]^ In another study, leveraging the capabilities to control the cellular components within the model, we investigated the interplay of immune cells on cancer cell invasive behavior.^[^ ^71^ ^]^ Furthermore, our group integrated the chip technology with single cell‐resolution studies to understand the influence of niche components on glioblastoma cancer.^[^ ^69b^ ^]^ These studies demonstrate the feasibility of the TMEC model in creating a physiologically relevant system for cancer research.

Building upon these foundational works, here, we report the development of a next‐generation organotypic breast TMEC model to decipher the complex interplay between CAFs and Mφs on TNBC progression. Specifically, we established a triculture invasion assay incorporating TNBC cells into the tumor region and CAFs and Mφs into the stromal region of the platform. The presence of both CAFs and Mφs exerted protumorigenic effects and aided in tumorigenesis. Our triculture invasion assay showed that the presence of CAFs and Mφs increased the migratory potential of the cancer cells but the effect was pronounced in triculture complementing the previous studies.^[^ ^27^, ^72^ ^]^ During the progression of cancer, reciprocal communication between CAFs and Mφs enhances cancer cell proliferation. Similarly, in our Tri condition, we observed an increased proliferation of cancer cells, confirming the supportive role of CAFs and Mφs in orchestrating tumor growth.^[^ ^73^ ^]^

Macrophages within the TME play an integral role in the support of cancer cell migration and invasion through bidirectional interactions with components within the TME. Particularly, Mφs are highly plastic cells that exert a double‐edged sword potential with either tumor‐supporting or tumor‐inhibiting role. Our results from qPCR analysis of Mφs revealed the co‐presence of both M1 and M2‐like macrophages with a mix of both canonical marker gene expressions (M1‐INOS, TNF‐α, and M2‐CD163, and CD206). A similar observation was reported in a study that characterized the tumor‐infiltrating immune components of human breast cancer.^[^ ^74^ ^]^ Furthermore, the polarization of Mφs can be triggered by the presence of stromal cells such as CAFs.^[^ ^75^ ^]^ We speculate that the presence of both tumor cells and CAFs may have a combinatorial effect on the functional state of the Mφs within our model. This highlights that Mφs exist in a spectrum of functional states within the TME further confirming the phenotypic diversity of these cell types with a unique expression profile.

To fully capture the precise interaction among tumor, CAF, and Mφ, we integrated our study with scRNA‐seq to mechanistically probe into the effect of CAF and Mφ in tumor progression. Before delineating the molecular mechanisms, we primarily validated the physiological relevance of our 3D organotypic model by comparing the transcriptomic changes of the cancer cells in 3D versus 2D. DEG analysis of cancer cells showed a clear shift of cancer populations in 3D from 2D with upregulation of DEGs. Importantly, pathway enrichment analysis showed upregulation of hallmark cancer pathways such as IL‐6, JAK STAT3, NOD‐like receptor, and inflammatory response, consistent with previous studies.^[^ ^76^ ^]^ Furthermore, assessment of transcriptomic changes of cancer cells in the presence and absence of CAFs and Mφs revealed the involvement of *KYNU* gene particularly in Tri condition. These findings on gene expression in Tri aligned well with the recent study that revealed the overexpression of *KYNU* in TNBC patients compared to other subtypes. Importantly, *KYNU* is an enzyme of the kynurenine pathway that plays a crucial role in immune evasion in the case of TNBC.^[^ ^77^ ^]^ Additionally, a study investigating the effect of TDO2‐AhR signaling provided more evidence of increased expression of the *KYNU* gene by TNBC cells.^[^ ^78^ ^]^ Another study evaluated the clinical characteristics of KYNU expression in breast cancer patients with publicly available datasets namely TCGA and METABRIC. Findings from this study revealed that high KYNU expression was implicated with pathological characteristics such as age, tumor size, and tumor stage. This study also revealed a positive correlation with immune‐response‐related genes, suggesting a significant role in immune modulation within the TME.^[^ ^79^ ^]^ Importantly, a comparison of identified DEGs to the patient database revealed a significant association between tryptophan catabolism and poorer overall survival. Tryptophan catabolism is an important metabolic pathway that has been associated with poorer patient survival, particularly through the activity of *KYNU* enzyme. A study by Xue et al. demonstrated that tryptophan catabolism contributes to the depletion of T cells, underscoring the impact on immune evasion by tumors.^[^ ^80^ ^]^ Another study indicated that *KYNU* could underpin the breast tumor cell invasion through the CD44 axis.^[^ ^81^ ^]^ Consistent with previous studies, findings from our metabolic flux analysis emphasize the potential of targeting and/or disrupting this metabolic pathway (tryptophan catabolism) through the *KYNU* gene thereby circumventing the immune evasion and improving the overall patient outcome.

Our investigation into the polarized state of Mφs revealed a distinct trajectories unique for each culture system (3D TMEC vs 2D). Analysis of macrophage genes showed relatively higher expression of the M2 gene (CD163) in Tri condition compared to CoMφ, confirming the presence of pro‐tumorigenic macrophages. Additionally, our findings from qPCR analysis validated the observed phenotypic characteristics of Mφs within our TMEC model. Furthermore, we also observed higher expression of certain relevant genes such as IL‐6 and MMP9 pertinent to the Tri conditions. IL‐6 expression by Mφs can influence both cancer cells and CAFs. Particularly, studies have reported that Mφ‐derived IL‐6 regulates tumor progression and enriches cancer stem cell population thereby contributing to treatment resistance and relapse.^[^ ^82^ ^]^ On the other hand, Mφ‐derived IL‐6 contribute to the proinflammatory phenotype of CAFs, which potentiates the oncogenic transformation of epithelial cells.^[^ ^26a^ ^]^ MMP‐9, a member of MMP family, has been reported to play an important role in matrix remodeling and invasion in breast cancer.^[^ ^83^ ^]^ Together, analysis of macrophage polarization state highlights the multifaceted role of cellular communication in determining the fate of macrophages. Furthermore, the presence of numerous cytokines could be attributed to the directed migration of cancer cells that were observed through our live‐imaging analysis. Importantly, we observed a continuum spectral state of Mφs within our model system through the scRNA‐seq analysis, as observed in patient tissue samples.^[^ ^74^ ^]^ It is essential to emphasize that these findings demonstrate the power of these models in capturing physiologically relevant readouts.

Analysis of tripartite communication of tumor, CAF, and Mφ through L–R analysis identified intriguing L–R pairs. Particularly, we observed the activation of CD47–SIRPA axis between Tri versus CoCAF, with the involvement of the *TNC* gene downstream. *TNC* gene encodes for the ECM protein tenascin C and has been shown to have several implications in cancer such as migration, invasion, proliferation, and immune evasion.^[^ ^26a^ ^]^ Markedly, in triculture, we observed similar behavior of cancer cells with increased migration potential and proliferative capabilities. Similarly, a comparison of Tri versus CoMφ revealed the involvement of downregulation of target genes such as *EGR1* and *ZFP36*, consistent to data obtained from TNBC patient tissue samples.^[^ ^63^ ^]^

Though the involvement of tryptophan catabolism in cancer is relatively known, our knowledge about *KYNU's* contribution to TNBC is scarce, including conflicting reports considering *KYNU* as an oncogenic suppressor.^[^ ^84^ ^]^ In our study, through metabolic flux analysis, we showed the impact of KYNU on the tryptophan pathway, demonstrating its heightened activity under Tri condition. To further validate the potential of targeting the identified pathway (tryptophan catabolism) from scRNA‐seq analyses, we utilized small‐molecule drug inhibition that could block the Trp/KP pathway within our TMEC model due to the upregulation of *KYNU* gene. Notably, the addition of the small molecule drug (Ro‐61‐8048) significantly inhibited the migration of cancer cells in Tri condition, emphasizing the importance of tryptophan catabolism as a potential drug target for potential clinical applications. In summary, combining our functional TMEC assays along with comprehensive bioinformatic analysis, we investigated novel mechanisms that drive invasion, tumorigenesis, and immune evasion involved in TNBC due to the presence of stromal and immune cells. Our study revealed several clinically relevant pathways, genes, and provided insights into the biology of the synergistic influence of CAFs, Mφs, and tumor cells using an in vitro organotypic system.

While our model successfully incorporates key stromal and immune components of the TME and provides valuable insights into the metabolic alterations of cancer cells within our TMEC model, further cellular components could still be added to fully encompass the complexity of in vivo interactions. For instance, adaptive immune cells such as T cells or dendritic cells, as well as vascularized compartments, could be added to the system. Additionally, while our model incorporates a biomimetic hydrogel matrix, our focus was not on assessing the role of ECM stiffness on tumor progression. A potential avenue could be variation of stromal ECM stiffness and conducting studies related to tumor invasion in the presence of immune and stromal cells. Building upon these key findings, our future work will focus on incorporating these additional cellular and matrix components to better replicate the complexities of the TME. It is noteworthy to mention that integrating such complex models with bioinformatic‐driven analyses will enable a more comprehensive understanding of TME interactions, thereby improving treatment outcomes and advancing our fundamental knowledge of cancer biology.

## 4 Conclusion 

The breast TME is heavily infiltrated with stromal CAFs and Mφs, which are strongly linked to poor prognosis. This underscores the pressing need for a deeper understanding of the mechanisms driving tumor growth. While many studies have emphasized the importance of investigating cancer and its surrounding environment, few have focused on mechanistic understanding of the synergetic impact of CAFs and Mφs in TNBC. In this study, we reported the establishment of an organotypic tumor‐on‐a‐chip model system, called the TMEC, that enabled the triculture of tumor cells, CAFs, and Mφs in a spatially organized manner, mimicking the in vivo organotypic TME. Using this model, we rigorously examined the influence of CAFs and Mφs on cancer cell migration, proliferation, morphological changes, and invasion. Our findings unequivocally revealed that CAFs and Mφs have a synergistic influence in driving cancer progression. Moreover, by integrating our model system with single‐cell resolution studies (scRNA‐seq), we uncovered the involvement of key cancer characteristics within in our 3D TMEC system. Specifically, through differential gene expression analysis, we unambiguously identified the upregulation of the *KYNU* gene, a key player in tryptophan catabolism and the KP, as a pivotal regulator in supporting tumor growth. Consistent with this, a comparison of the identified DEGs with patient datasets revealed a significant association between tryptophan catabolism and clinical outcome, underscoring the critical involvement of this pathway in determining patient prognosis. Importantly, pharmacological inhibition of kynurenine pathway using small molecule inhibitor Ro 61‐8048 blunted the invasion abilities of the cancer cells. Overall, our findings corroborate our model's robustness in recapitulating the complex processes within the TME and unveiling key biological pathways that govern the tumor progression. The proposed model could be potentially applied for patient‐specific biological studies as well as drug screening and target discoveries.

## 5 Experimental Section

### Microfluidic (TMEC) Design Features and Fabrication

The TMEC platform utilized for understanding cellular communications consisted of three distinctive regions: an inner tumor region (red) and a central semicircular stroma region (green), bordered by flanking media channels on either side (Figure 1A). The diameter of the concentric regions was 3 mm, and the distance between the edge of the tumor to the stroma region was about 1 mm, which was the maximum distance available for the cells to migrate from the tumor region. To achieve spatial organization and to allow cellular communication between regions, trapezoidal microposts spaced at a distance of 100 µm bounded the interface of tumor–stroma and stroma–media regions. The presence of microposts allowed for the retention of different types of hydrogels into separate regions due to the contact angle of the hydrogel solution being supplementary to the microposts. All design features were carried out in AutoCAD and transferred to a transparent mask for further processing.

TMEC devices were fabricated using the standard photolithography and soft lithography techniques, similar to the previous works.^[^ ^23^, ^69^, ^70^ ^]^ Briefly, SU‐8 3025 (Microchem, USA) negative photoresist was spin‐coated onto a 4″ silicon wafer to achieve an overall thickness of about 200 µm. Post baking, the wafer was overlaid with a transparent mask and exposed to two cycles of UV to transfer the device pattern onto the wafer (master mold). Furthermore, the wafer was developed, and its surface was silanized using methyl trichlorosilane (MTCS) to prevent the sticking of polydimethylsiloxane (PDMS) during device fabrication. For the soft lithography‐based microfluidic device fabrication, PDMS base (Sylgard 184 Silicon Elastomer Kit, Dow Corning) and curing agents were mixed (10:1 ratio), degassed, and cured at 80 °C for 1.5 h. Cured PDMS was peeled off the wafer; then, inlet and outlet ports were created using 1 and 1.5 mm biopsy punch, respectively, and cut into individual devices. Devices were bonded to a glass coverslip (18 × 18 mm) through oxygen plasma treatment (Harrick Plasma, USA) and sterilized through two rounds of autoclaving. Post‐autoclaving devices were dehydrated overnight at 80 °C before culturing the cells (breast TME matrix).

### Cell Culture and Macrophage Differentiation

Sum159 cells (University of Arizona), stably transduced to express m‐Cherry protein, were cultured in F12 media supplemented with 5% heat‐inactivated fetal bovine serum (HI‐FBS), 1% penicillin–streptomycin, 1 µg mL^−1^ hydrocortisone, and 5 µg mL^−1^ insulin. Patient‐derived CAFs from breast tumor tissue samples (age: 82, type: invasive cancer; tumor size: 2.6 cm, stage 3, receptor: triple negative) were isolated upon receiving proper approval from the Institutional Review Board (IRB 2130‐00) at Mayo Clinic of Arizona. Isolation of patient‐derived CAFs was conducted in compliance with the ethical guidelines outlined in the Declaration of Helsinki, the U.S. Common Rule, the Belmont Report, and CIOMS. Informed consent was obtained from all patients at the Mayo Clinic. All patient information was de‐identified prior to use in the laboratory at ASU. CAF cells were established following the previous protocol.^[^ ^23^ ^]^ Briefly, tissues containing CAFs were digested overnight using a solution containing collagenase I (1 mg mL^−1^, Worthington) and hyaluronidase (125 U mL^−1^, Sigma–Aldrich) and 10% FBS at 37 °C. Following this, cells were strained using a cell strainer and cultured in dulbecco's modified eagle medium (DMEM) supplemented with 10% HI‐FBS, 1% penicillin–streptomycin, and 1% l‐glutamine, similar to the previous publication.^[^ ^23^ ^]^ Both Sum159 and CAFs were cultured in a T75 cm^2^ flask and subcultured using 0.05% trypsin ethylenediaminetetraacetic acid (trypsin‐EDTA) up to passages 30 and 10, respectively.

THP‐1 (ATCC) monocytic cells were utilized to model naïve Mφs. THP‐1 cells were cultivated in RPMI media supplemented with 10% HI‐FBS, 1% penicillin–streptomycin, and 1% l‐glutamine. Cells were cultured as suspensions in a T75 cm^2^ flask prior to differentiation and used up to passage 15. To initiate differentiation of monocytes to macrophage, 1 million cells per well of monocytes were seeded in a 6‐well plate and incubated with RPMI media supplemented with 50 ng mL^−1^ PMA for 24 h. Post differentiation, cells were rested for 24 h with RPMI media (without PMA). After 48 h, cells were dissociated from the well plate using Accutase (Gibco) and were used for the establishment of the 3D TMEC model. All cells were maintained at optimal culture conditions of 37 °C, 5% CO_2_ inside humidified cell culture incubators.

### Microfluidic Construction of In Vitro Breast TME

Before the injection of cells into the platform, fabricated devices were surface‐treated following the established protocol.^[^ ^71^ ^]^ Briefly, devices were incubated with poly‐l‐lysine for 1.5 h at 37 °C. Next, devices were washed once with deionized (DI) H_2_O followed by incubation of devices with 1% (v/v) glutaraldehyde at room temperature (RT) for 2 h. Finally, devices were washed thrice with DI H_2_O and stored at 80 °C overnight before using them for experiments.

For the reconstruction of multicellular breast TME, tumor and stroma regions of the devices were filled with cancer, fibroblasts, and immune cells, respectively. To establish a cancer‐cell‐rich tumor region, Sum159 cells at a density of 10 × 10^6^ cells mL^−1^ were resuspended in 1:1 of Matrigel and collagen‐I hydrogel with a final concentration of collagen‐I being 1 mg mL^−1^. Prepared cell:hydrogel mixture was injected into the tumor region of the sterilized device. To initiate the crosslinking of the hydrogel, devices were placed at 37 °C for 4 min and flipped every minute to achieve a homogeneous distribution of cells. Devices were placed inside a large Petri dish filled with DI water at all times to maintain a humidified environment.

After polymerization of the tumor region, the stroma region was injected depending on the culture conditions. In the case of Mono, acellular 2 mg mL^−1^ collagen I hydrogel solution was injected into the stroma region and crosslinked for 8 min without flipping. For co‐and triculture conditions (CoCAF, CoMφ, and Tri), CAFs and/or Mφs were mixed with collagen‐I with cell densities of 25 × 10^4^ and 5 × 10^5^ cells mL^−1^, respectively, and a collagen‐I concentration of 2 mg mL^−1^ (Table S1, Supporting Information). The stromal and immune cell numbers within the model were carefully optimized to mimic the in vivo cell concentration.^[^ ^85^ ^]^ Specifically, the concentration of CAFs was adjusted from previous publications to include additional cells in the same stromal region. This reduction in CAF concentration eased overcrowding in the stromal region and also prevented the gel from peeling. Mφs that account for 5–50%^[^ ^86^ ^]^ of tumor mass were incorporated at a density of 5%, ensuring that their concentration did not overcrowd the stromal region. Then, the cell:hydrogel mixture was carefully injected into the stroma region of the devices and allowed to crosslink for 8 min while flipping every 2 min to achieve a homogeneous mixture of cells within the platform. Finally, devices were cultured using F12 media, and the media were exchanged every day.

### Analysis of Cancer Cell Migration

To characterize the migratory behavior of tumor cells in the presence of different stromal and immune components, phase‐contrast images of the devices were obtained from day 0 through day 3 using a Zeiss Axio Observer Z1 microscope. On day 3, the migration distance of the cancer cells (red) was quantified by measuring the farthest migrated cancer cells in the stroma region. All the migration distance quantification was performed using NIH ImageJ. To delineate the influence of CAFs and Mφs, migration distance was normalized to the Mono condition.

### Time‐Lapse Imaging and Microscopy

Fluorescent time‐lapse imaging was obtained using the Zeiss Axio Observer Z1 microscope equipped with apotome2 and ZenPro software. After 48 h of interaction of various cells within the TOC model, time‐lapse videos of 16 h were recorded. All videos were captured overnight at 10× magnification and with a time interval of 45 min. Throughout the procedure, samples were maintained at optimal cell culture conditions (37 °C and 5% CO_2_) using a custom‐built incubation chamber that was attached to the microscope system. Quantification of migration metrics of different cell types was performed using a manual tracking plugin within NIH ImageJ.

### IF Staining

After 72 h of culture, all samples were washed once with 1× phosphate buffered saline (PBS) and fixed with 4% paraformaldehyde (PFA) for 30 min at 37 °C. Following the fixation, samples were rinsed 2× with PBS–glycine for 10 min at RT. After PBS–glycine wash, devices were washed once with PBS–Tween‐20 ((PBS–polyoxyethylene (20) sorbitan monolaurate) (0.05% (v/v) Polyoxyethylene (20) sorbitan monolaurate in PBS) for 10 min at RT. Then, cells were permeabilized with 0.1% Triton‐X‐100 for 30 min at RT and blocked with 10% goat serum (diluted with PBS–Tween‐20) for 1 h at RT.

To stain the cells for actin cytoskeleton and nuclei, Alexa Fluor 488–Phalloidin (1:1000) and 4′,6‐diamidino‐2‐phenylindole (DAPI) (1:1000) diluted in PBS–Tween 20 was added to the samples and incubated overnight at 4 °C. The next day, devices were washed 3× with PBS–Tween‐20 for 15 min each. After washing all samples, PBS–Tween‐20 was replaced with 1× PBS and preserved at 4 °C until imaging.

For IF staining with Ki‐67 (1:100, Cell Signaling Technology), samples were washed twice with PBS–Tween for 10 min at RT. Following the washing, samples were permeabilized with IF buffer for 30 min at RT and blocked using 10% goat serum for 1 h at RT. Conjugated Ki‐67 antibody was diluted in 10% goat serum, added to the samples, and incubated overnight at 4 °C. Next day samples were counterstained with 1:1000 DAPI and incubated for 1 h at RT. After counterstaining, samples were washed 3–5 times with PBS–Tween. Finally, 1× PBS was added to the devices before imaging. All fluorescence images were obtained using a Zeiss Axio Observer Z1 microscope equipped with apotome2 at 10× and 20× magnification. *Z*‐stacked images were obtained and reconstructed using FIJI, a version of ImageJ.

### Quantitative Real‐time Reverse Transcription‐PCR (qRT‐PCR)

To extract the cells from the device for gene expression analysis, devices were first washed with 1× PBS and then incubated with prewarmed collagenase (2 mg mL^−1^) for 30–35 min at 37 °C to digest the ECM. Cell suspension from multiple devices was pooled for each condition. To maximize the cell collection, devices were washed thrice with 1× PBS. Collected cells were centrifuged at 1200 rpm for 5 min and the supernatant was removed. Total RNA was extracted using the Total RNA Microprep kit (Zymo) following the manufacturer's protocol. RNA quality and concentration were assessed either with High Sensitivity RNA Tapes and/or a nanodrop spectrophotometer system. Complementary deoxyribonucleic acid (cDNA) was synthesized from Total RNA using iScript Reverse Transcriptase Supermix (QuantBio). iTaq Universal SYBR Green Supermix (BioRad) was used to perform qPCR on synthesized cDNA, with glyceraldehyde‐3‐phosphate dehydrogenase (GAPDH) as the housekeeping gene. Primers were validated via melt curve. For qPCR, 8 µm dilution of forward and reverse primers was used for 10 µL reactions within 96‐well plates, with 0.1 µL of cDNA per well. qPCR plates were analyzed with qTower 2.0. Primer sequences used for qRT‐PCR are provided in Table S6 (Supporting Information).

### scRNA‐Seq Sample Preparation

For scRNA‐seq, Sum159, CAFs, and Mφs were cultured in standard 2D culture plastics as well as in the 3D TMEC model (Table S1, Supporting Information). Cells from the devices were extracted using prewarmed collagenase I, as described above. Cells from multiple devices for each condition were pooled together and centrifuged. For the 2D cell extraction, TrypLE was used for detaching the cells from culture dishes and centrifuged. After centrifugation, samples were prepared for scRNA‐seq following the Parse (Version 1.1) protocol. Briefly, samples were barcoded using the Parse split and pool technique. After barcoding, RNA concentration was measured on High Sensitivity RNA Tape station and sequenced by Illumina NextSeq2000 at the Tgen (ASU) with an average read count per sample of 105 541 963.

### Preprocessing of scRNA‐seq Data

The raw sequencing data obtained from the experiments were preprocessed using the pipeline supplied by Parse Biosciences (v1.1.1). The reference genome and annotation files used in the data processing were GRCh38 and Ensemble 110. The function “ReadParseBio” in Seurat (v4.4.0) was used to load the count matrix in the output of Parse Biosciences pipeline.^[^ ^87^ ^]^

### Quality Control and Data Normalization

Genes that were detected in at least 12 cells were kept in the matrix. The following criteria were used to filter out cells: 1) total counts per cell < 125 000; 2) cells that have <35% mitochondrial counts. Pseudogenes and mitochondrial genes were removed from the matrix. For the remaining 9682 cells after filtering, the cell cycle scores were calculated using the function CellCycleScoring. The filtered count matrix was normalized using the function SCTransform to mitigate technical variability across cells. Furthermore, to minimize technical variability among samples, mitochondrial mapping percentage and cell cycle scores were as considered confounding sources of variation and were adjusted during the normalization process.

### Doublet Detection and Exclusion

DoubletFinder^[^ ^88^ ^]^ was used to identify doublets in each well individually. The expected doublet rate was set to 3%, and the top 50 principal components (PCs) were utilized for single‐cell detection. All other parameters were kept at their default settings. After detecting doublets in each well, the results were combined into a single Seurat object. From a total of 12 000 cells, 100 were labeled as doublets, while the remaining 11 900 were classified as singlets. The Seurat object, after excluding the doublet‐labeled cells, was used for the downstream analyses.

### Cell Clustering and Batch Correction

Using Seurat, all remaining genes in the normalized matrix were used to perform PC analysis (PCA). At a resolution of 0.1, cell clustering analysis was performed using the first 50 principal components, resulting in a total of 6 clusters. UMAP was employed to visualize the high‐dimensional data in two dimensions.^[^ ^89^ ^]^ The cell type of each cluster was determined based on the average expression levels of marker genes within each cluster.

Harmony^[^ ^90^ ^]^ was used to correct for the possible batch effect between 2D and 3D cultures. Clustering was performed using the first 50 corrected PCAs, and cells were similarly divided into 6 clusters at 0.1 resolution.

For the analysis of cancer cells across different culture conditions or treatments, the NormalizeData, FindVariableFeatures, and ScaleData functions were used for normalization, with mitochondrial mapping percentage and cell cycle scores regressed out. Clustering was performed using the first 50 PCs. At a resolution of 0.08, cancer cells from 2D and 3D cultures were divided into four clusters after batch effect correction using Harmony. At a resolution of 0.09, cancer cells from different treatments in the 3D culture were grouped into three clusters.

### Differential Gene Expression and Pathway Enrichment Analysis

Differential expression (DE) analysis between 2D and 3D cancer cells was performed by the function FindMarkers using the *t*‐test and the Wilcoxon rank‐sum test. The normalized data from the RNA assay in the Seurat object were used. DEGs were identified with the adjusted *p*‐value of <1e^−10^.

Another approach for DE analysis is pseudo‐bulk DE analysis. The raw counts of cancer cells in the scRNA‐seq data were aggregated based treatments. The resulting count matrix was filtered to remove lowly expressed genes and mitochondrial genes, followed by trimmed mean of M‐values (TMM) normalization. The biological coefficient of variation (BCV) was set to 0.4, and DE analysis was performed using the exactTest function in edgeR.^[^ ^91^ ^]^

All genes were sorted in descending order based on the log2 fold‐change (log2FC) values, and the GSEA^[^ ^92^ ^]^ was then performed on 5870 pathway/ontology terms in KEGG, Molecular Signatures Database^[^ ^93^ ^]^ (MSigDB: hallmark gene sets, curated gene sets, regulatory target gene sets, ontology gene sets, and oncogenic signature gene sets), and GO databases using this preranked gene list as input.

The data slot from the RNA assay in the Seurat object were extracted for ssGSEA analysis using GSEApy.^[^ ^94^ ^]^ The gene sets used for ssGSEA included KEGG, WikiPathways, GOBP, and Hallmark gene sets. The resulting NES matrix was grouped based on the condition of each cell, and a *t*‐test or a Wilcoxon rank‐sum test was performed for each gene set.

For GO enrichment analysis, the genes were ranked in descending order based on the log2FC obtained from the DE analysis as the ranked list. GO enrichment analysis was performed using the gseGO function from ClusterProfiler.^[^ ^95^ ^]^ The resulting GO terms were then processed with the simplify function to remove redundant terms.

### Patient Survival Analysis

Expression data from TCGA and Metabric were obtained, and ssGSEA was performed on each sample of the GSEApy package. For each gene set term, patients were ranked by their NES in descending order. The top 20% of patients with the highest NES values were designated as the “high” group, while the bottom 20% with the lowest NES values were designated as the “low” group for that term. The survival rates of the high and low groups for each gene set term were compared using Kaplan–Meier analysis implemented in the survminer *R* package.^[^ ^96^ ^]^

### Macrophage Clustering and Batch Correction

Cells identified as macrophages were selected for further analysis. Preprocessing was performed using the NormalizeData and ScaleData functions, with the vars.to.regress parameter in ScaleData set to mitochondrial mapping percentage and cell cycle scores and the features parameter was set to the macrophage marker gene list. PCA was then conducted using the macrophage marker gene list, followed by batch effect correction between 2D and 3D cultures using Harmony. Clustering was performed using the first 15 corrected PCs, and cells were divided into four clusters at a resolution of 0.8.

### Identification of Macrophage Trajectory Pattern

Slingshot^[^ ^97^ ^]^ was used to infer the trajectory and pseudotime of macrophages. The slingshot function was applied with the reducedDim parameter set to UMAP, clusterLabels set to Cluster ID, and start.clus set to Cluster 4. As a result, a primary cell lineage was identified.

Similarly, another approach for trajectory and pseudotime inference was performed using Monocle3.^[^ ^98^ ^]^ The root_cells parameter was set to Cluster 4, and the analysis similarly identified a primary cell lineage. The Pearson correlation coefficient was calculated between the pseudotime inferred from Slingshot and gene expression levels. GSEA was then performed using the TFT:TFT_LEGACY (legacy transcription factor targets) database.

### Ligand–Receptor Pair and Cell–Cell Interaction

Cell–cell communication analysis was performed by Nichenet.^[^ ^53^ ^]^ Among the cell types identified from the single‐cell data, Sum159 was defined as the receiver and CAF/Mφ was defined as the sender. Additionally, to study the autocrine signaling, Sum159 itself was also treated as the sender. By comparing the expression level differences between Tri and CoCAF/CoMφ for genes in the receiver and sender, NicheNet identifies L–R pairs and predicted targets.

For each identified L–R pair, the sum of the log2 fold changes in expression levels of the ligand and receptor was used as the weight of the pair. The L–R pairs were sorted in descending order based on the absolute value of the weights. The sum of the weights for all L–R pairs corresponding to each ligand was calculated and ranked in descending order based on absolute values. The top ten ligands were selected and visualized in a chord diagram.

For the top five ligand–receptor pairs identified for each sender, ligand–receptor–target signaling networks were constructed using the predicted targets from NicheNet and the public data sources utilized by NicheNet. The network was visualized in a Sankey diagram.

Another tool used to predict cell–cell communication was CellChat. First, preprocessing was performed separately for Tri, CoCAF, and CoM using CellChat. When comparing Tri with CoCAF/CoM, Tri's cell types were filtered to ensure consistency with the cell types present in CoCAF/CoM.

Following the standard CellChat^[^ ^54^ ^]^ workflow, the identifyOverExpressedGenes function was applied with thresh.fc set to 0 and thresh.p set to 1 to identify L–R pairs. The identified L–R pairs with a *p*‐value of <0.05 were ranked in descending order based on their probability (prob), and the top 20 candidate ligands were selected. The L–R pairs corresponding to these candidate ligands were processed using the same approach as described for NicheNet and visualized in a chord diagram.

### Metabolic Flux Inference

METAFlux was used to infer metabolic fluxes. First, the raw counts of 3D‐cultured cancer cells from scRNA‐seq data were aggregated based on treatment. The resulting count matrix was filtered to remove lowly expressed genes and mitochondrial genes, followed by TMM normalization. Metabolic flux inference was then performed following the standard bulk analysis workflow, and pathway‐level activity was computed for 142 pathways. Pathway activity was defined as the average flux of reactions associated with the given pathway.

Subsequently, single‐cell analysis was performed following the standard workflow, with the n_bootstrap parameter in the calculate_avg_exp function set to 100. Reactions associated with KYNU and KMO were extracted, and a box plot was generated to visualize the bootstrap distribution, showing the differences in metabolic fluxes among cancer cells under different treatments. A two‐sided Games–Howell test was used to compare Tri with other conditions. The *p*‐values were adjusted using the Holm correction method.

### Inhibition of Identified Metabolic Pathway through Small Molecule Drug

To show Trp catabolism or KP as a potential target for cancer therapy, a small molecule inhibitor namely (Ro‐61‐8048) that targets the metabolic enzymes *KMO* and *KYNU* was used. To assess the effect of the drug five concentrations of the drug (5, 10, 20, 50, and 100 µm) were screened based on previous studies. All drugs were diluted in Sum159 media and concentrations used were within the half‐maximal inhibitory concentration (IC_50_) values for this drug. Particularly for this study, two experimental conditions, namely the Mono and Tri conditions, were focused. Devices were injected with respective cell types for each condition similar to other assays. For creating the treatment regimen, cells were initially allowed to interact within the TMEC model for about 48 h, and on day 2 of the invasion assay, cells were replaced with media containing the respective concentration of the drug. In the case of control (no drug treatment), cells were added with media (Sum159 media) containing 0.4% dimethyl sulfoxide. After the addition of the drug, the cells were incubated with the drug for 48 h. The duration after the addition of the drug to the model was referred to as post‐treatment and devices were imaged on the end of 48 h.

### Viability Assay

The viability of the cells within the microfluidic model was assessed at 48 h post‐drug treatment to comprehend the impact of the drug compound on the immune, stromal, and tumor cells. Specifically, to assess this, two monoculture conditions namely, Mono‐CAF and Mono‐Mφ, were additionally included to delineate the influence of the drug on the stromal and immune cells. Devices were washed once with 1× PBS, then incubated with 2 µm of Calcein AM and 4 µm of ethidium homodimer‐III (EthD‐III) for 30 min at 37 °C. Post‐staining devices were washed twice with 1× PBS and the devices were incubated with fresh 1× PBS solution and imaged with the microscope.

### Statistical Analysis

For all experiments conducted, values were obtained from a minimum of three biological replicates (*n* > 3), each with at least four technical replicates. All reported data were presented as dot plots overlaid on a bar chart expressed as average ± standard deviation. Statistical analysis was performed using GraphPad Prism Software (Version 10, GraphPad Software Inc., CA). The data were compared using paired *t*‐test, one‐way analysis of variance (ANOVA), and two‐way ANOVA followed by multiple comparisons across different groups. UMAP and heat maps were generated using custom *R* scripts.

## Conflict of Interest

The authors declare no conflict of interest.

## Author Contributions

K.R. wrote the manuscript with input from M.N. J.G.P., K.R., and M.N. conceptualized and designed all the experiments. B.P. provided the patient‐derived CAFs for this project. K.R. and L.S. performed the scRNA extraction of cells from the TOC platform. J.G.P. and Y.Z. performed the bioinformatic analysis on sc‐RNA sequencing data. K.R., L.S., Y.Z., J.G.P., and M.N. contributed to the sc‐RNA data interpretation. T.J.M.M. contributed to the analysis of real‐time migration assay. All authors reviewed the manuscript.

## Supporting information



Supporting Information

Supplemental Movie 1

Supplemental Movie 2

Supplemental Movie 3

Supplemental Movie 4

Supplemental Movie 5

Supplemental Movie 6

Supplemental Movie 7

Supplemental Movie 8

Supporting Information

Supporting Information

Supporting Information

## Data Availability

The data that support the findings of this study are available from the corresponding author upon reasonable request.

## References

[1] N. M. Anderson , M. C. Simon , Curr. Biol. 2020, 30, R921. 32810447 10.1016/j.cub.2020.06.081 PMC8194051

[2] R. Baghban , L. Roshangar , R. Jahanban‐Esfahlan , K. Seidi , A. Ebrahimi‐Kalan , M. Jaymand , S. Kolahian , T. Javaheri , P. Zare , Cell Commun. Signaling 2020, 18, 59. 10.1186/s12964-020-0530-4 PMC7140346 32264958

[3] I. S. Chan , A. J. Ewald , J. Clin. Invest. 2022, 132, e143762. 35289318 10.1172/JCI143762 PMC8920322

[4] M. De Palma , D. Biziato , T. V. Petrova , Nat. Rev. Cancer 2017, 17, 457. 28706266 10.1038/nrc.2017.51

[5] a) L. Bejarano , M. J. C. Jordāo , J. A. Joyce , Cancer Discovery 2021, 11, 933; 33811125 10.1158/2159-8290.CD-20-1808

[6] D. F. Quail , J. A. Joyce , Nat. Med. 2013, 19, 1423. 24202395 10.1038/nm.3394 PMC3954707

[7] M. Hu , L. Huang , Adv. Drug Delivery Rev. 2022, 183, 114137. 10.1016/j.addr.2022.114137 35143893

[8] K. E. de Visser , J. A. Joyce , Cancer Cell 2023, 41, 374. 36917948 10.1016/j.ccell.2023.02.016

[9] A. J. Oliver , P. K. H. Lau , A. S. Unsworth , S. Loi , P. K. Darcy , M. H. Kershaw , C. Y. Slaney , Front. Immunol. 2018, 9, 70. 29445373 10.3389/fimmu.2018.00070 PMC5797771

[10] H. Salmon , R. Remark , S. Gnjatic , M. Merad , Nat. Rev. Cancer 2019, 19, 215. 30867580 10.1038/s41568-019-0125-9 PMC7787168

[11] B. C. Özdemir , T. Pentcheva‐Hoang , J. L. Carstens , X. Zheng , C.‐C. Wu , T. R. Simpson , H. Laklai , H. Sugimoto , C. Kahlert , S. V. Novitskiy , Cancer Cell 2014, 25, 719. 24856586 10.1016/j.ccr.2014.04.005 PMC4180632

[12] L. Bonapace , M.‐M. Coissieux , J. Wyckoff , K. D. Mertz , Z. Varga , T. Junt , M. Bentires‐Alj , Nature 2014, 515, 130. 25337873 10.1038/nature13862

[13] a) A. Tiwari , R. Trivedi , S.‐Y. Lin , J. Biomed. Sci. 2022, 29, 83; 36253762 10.1186/s12929-022-00866-3 PMC9575280

[14] F. Xing , J. Saidou , K. Watabe , Front. Biosci. 2010, 15, 166. 10.2741/3613 PMC2905156 20036813

[15] a) D. Yang , J. Liu , H. Qian , Q. Zhuang , Exp. Mol. Med. 2023, 55, 1322; 37394578 10.1038/s12276-023-01013-0 PMC10394065

[16] K. Shiga , M. Hara , T. Nagasaki , T. Sato , H. Takahashi , H. Takeyama , Cancers 2015, 7, 2443. 26690480 10.3390/cancers7040902 PMC4695902

[17] M. J. Pittet , O. Michielin , D. Migliorini , Nat. Rev. Clin. Oncol. 2022, 19, 402. 35354979 10.1038/s41571-022-00620-6

[18] a) D. J. Kloosterman , L. Akkari , Cell 2023, 186, 1627; 36924769 10.1016/j.cell.2023.02.020

[19] a) Y. Pan , Y. Yu , X. Wang , T. Zhang , Front. Immunol. 2020, 11, 583084; 33365025 10.3389/fimmu.2020.583084 PMC7751482

[20] V. Brancato , J. M. Oliveira , V. M. Correlo , R. L. Reis , S. C. Kundu , Biomaterials 2020, 232, 119744. 31918229 10.1016/j.biomaterials.2019.119744

[21] B. Pinto , A. C. Henriques , P. M. A. Silva , H. Bousbaa , Pharmaceutics 2020, 12, 1186. 33291351 10.3390/pharmaceutics12121186 PMC7762220

[22] J. Komen , S. M. van Neerven , A. van den Berg , L. Vermeulen , A. D. van der Meer , EBioMedicine 2021, 66, 103303. 33773183 10.1016/j.ebiom.2021.103303 PMC8024912

[23] D. D. Truong , A. Kratz , J. G. Park , E. S. Barrientos , H. Saini , T. Nguyen , B. Pockaj , G. Mouneimne , J. LaBaer , M. Nikkhah , Cancer Res. 2019, 79, 3139. 30992322 10.1158/0008-5472.CAN-18-2293 PMC6664809

[24] M. J. Carroll , K. C. Fogg , H. A. Patel , H. B. Krause , A.‐S. Mancha , M. S. Patankar , P. S. Weisman , L. Barroilhet , P. K. Kreeger , Cancer Res. 2018, 78, 3560. 29739756 10.1158/0008-5472.CAN-17-3341 PMC6030435

[25] S. Mi , Z. Liu , Z. Du , X. Yi , W. Sun , Biotechnol. Bioeng. 2019, 116, 1731. 30802293 10.1002/bit.26961

[26] a) X. Mao , J. Xu , W. Wang , C. Liang , J. Hua , J. Liu , B. Zhang , Q. Meng , X. Yu , S. Shi , Mol. Cancer 2021, 20, 131; 34635121 10.1186/s12943-021-01428-1 PMC8504100

[27] B. Gok Yavuz , G. Gunaydin , M. E. Gedik , K. Kosemehmetoglu , D. Karakoc , F. Ozgur , D. Guc , Sci. Rep. 2019, 9, 3172. 30816272 10.1038/s41598-019-39553-z PMC6395633

[28] a) A. Labernadie , T. Kato , A. Brugués , X. Serra‐Picamal , S. Derzsi , E. Arwert , A. Weston , V. González‐Tarragó , A. Elosegui‐Artola , L. Albertazzi , J. Alcaraz , P. Roca‐Cusachs , E. Sahai , X. Trepat , Nat. Cell Biol. 2017, 19, 224; 28218910 10.1038/ncb3478 PMC5831988

[29] R. Sato , K. Imamura , T. Semba , Y. Tomita , S. Saeki , K. Ikeda , Y. Komohara , M. Suzuki , T. Sakagami , H. Saya , Y. Arima , Cancer Res. 2021, 81, 4751. 34289987 10.1158/0008-5472.CAN-20-3941 PMC9397619

[30] L. Prasmickaite , E. M. Tenstad , S. Pettersen , S. Jabeen , E. V. Egeland , S. Nord , A. Pandya , M. H. Haugen , V. N. Kristensen , A.‐L. Børresen‐Dale , Oslo Breast Cancer Research Consortium (OSBREAC) , O. Engebråten , G. M. Mælandsmo , Mol. Oncol. 2018, 12, 1540. 29741811 10.1002/1878-0261.12319 PMC6120223

[31] W. Liu , M. Zhu , X. Li , L. Er , S. Li , Dis. Markers 2023, 2023, 9226712. 36817086 10.1155/2023/9226712 PMC9934984

[32] N. Takasaka , R. I. Seed , A. Cormier , A. J. Bondesson , J. Lou , A. Elattma , S. Ito , H. Yanagisawa , M. Hashimoto , R. Ma , JCI Insight 2018, 3, e122591. 30333313 10.1172/jci.insight.122591 PMC6237456

[33] a) Y. Tokumaru , M. Oshi , E. Katsuta , L. Yan , V. Satyananda , N. Matsuhashi , M. Futamura , Y. Akao , K. Yoshida , K. Takabe , Am. J. Cancer Res. 2020, 10, 897; 32266098 PMC7136911

[34] R. Kerslake , B. Belay , S. Panfilov , M. Hall , I. Kyrou , H. S. Randeva , J. Hyttinen , E. Karteris , C. Sisu , Cancers 2023, 15, 3350. 37444459 10.3390/cancers15133350 PMC10340606

[35] a) B. Heng , A. A. Bilgin , D. B. Lovejoy , V. X. Tan , H. H. Milioli , L. Gluch , S. Bustamante , T. Sabaretnam , P. Moscato , C. K. Lim , G. J. Guillemin , Breast Cancer Res. 2020, 22, 113; 33109232 10.1186/s13058-020-01351-1 PMC7590459

[36] J. L. Harden , S. M. Lewis , S. R. Lish , M. Suárez‐Fariñas , D. Gareau , T. Lentini , L. M. Johnson‐Huang , J. G. Krueger , M. A. Lowes , J. Allergy Clin. Immunol. 2016, 137, 1830. 26725996 10.1016/j.jaci.2015.09.055 PMC4899291

[37] R. Schwarcz , T. W. Stone , Neuropharmacology 2017, 112, 237. 27511838 10.1016/j.neuropharm.2016.08.003 PMC5803785

[38] C. Güngör , C. Kondziella , B. Mercanoglu , G. Wolters‐Eisfeld , J. R. Izbicki , M. Bockhorn , C. Schröder , HPB 2019, 21, S866.

[39] K. Hushmandi , B. Einollahi , S. H. Saadat , E. H. C. Lee , M. R. Farani , E. Okina , Y. S. Huh , N. Nabavi , S. Salimimoghadam , A. P. Kumar , Mol. Metab. 2024, 84, 101952. 38705513 10.1016/j.molmet.2024.101952 PMC11112377

[40] A. Masjedi , V. Hashemi , M. Hojjat‐Farsangi , G. Ghalamfarsa , G. Azizi , M. Yousefi , F. Jadidi‐Niaragh , Biomed. Pharmacother. 2018, 108, 1415. 30372844 10.1016/j.biopha.2018.09.177

[41] L. E. Navas , A. Carnero , Signal Transduction Targeted Ther. 2021, 6, 2. 10.1038/s41392-020-00354-w PMC7775471 33384409

[42] A. N. Mogol , A. Z. Kaminsky , D. J. Dutton , Z. Madak Erdogan , Endocrinology 2024, 165, bqae043. 38565429 10.1210/endocr/bqae043

[43] J. Cornice , D. Verzella , P. Arboretto , D. Vecchiotti , D. Capece , F. Zazzeroni , G. Franzoso , Genes 2024, 15, 197. 38397187 10.3390/genes15020197 PMC10888451

[44] S. K. Biswas , C. E. Lewis , J. Leukocyte Biol. 2010, 88, 877. 20573802 10.1189/jlb.0310153

[45] a) Y. Zhang , F. Zhong , L. Liu , Breast Cancer Res. 2024, 26, 129; 39232806 10.1186/s13058-024-01887-6 PMC11373130

[46] K. L. Spiller , E. A. Wrona , S. Romero‐Torres , I. Pallotta , P. L. Graney , C. E. Witherel , L. M. Panicker , R. A. Feldman , A. M. Urbanska , L. Santambrogio , G. Vunjak‐Novakovic , D. O. Freytes , Exp. Cell Res. 2016, 347, 1. 26500109 10.1016/j.yexcr.2015.10.017

[47] C. Melani , S. Sangaletti , F. M. Barazzetta , Z. Werb , M. P. Colombo , Cancer Res. 2007, 67, 11438. 18056472 10.1158/0008-5472.CAN-07-1882 PMC2646404

[48] Y.‐C. Chen , C.‐C. Chen , R.‐F. Chen , H.‐H. Chen , P.‐M. Chen , Curr. Issues Mol. Biol. 2024, 46, 13499. 39727934 10.3390/cimb46120806 PMC11674533

[49] M. Rakina , I. Larionova , J. Kzhyshkowska , Heliyon 2024, 10, e28332. 38571605 10.1016/j.heliyon.2024.e28332 PMC10988020

[50] a) J. Wang , N. Zhu , X. Su , Y. Gao , R. Yang , Front. Immunol. 2024, 14, 1264774; 38347955 10.3389/fimmu.2023.1264774 PMC10859433

[51] E. Timperi , P. Gueguen , M. Molgora , I. Magagna , Y. Kieffer , S. Lopez‐Lastra , P. Sirven , L. G. Baudrin , S. Baulande , A. Nicolas , G. Champenois , D. Meseure , A. Vincent‐Salomon , A. Tardivon , E. Laas , V. Soumelis , M. Colonna , F. Mechta‐Grigoriou , S. Amigorena , E. Romano , Cancer Res. 2022, 82, 3291. 35862581 10.1158/0008-5472.CAN-22-1427

[52] H. Zhao , L. Wu , G. Yan , Y. Chen , M. Zhou , Y. Wu , Y. Li , Signal Transduction Targeted Ther. 2021, 6, 263. 10.1038/s41392-021-00658-5 PMC8273155 34248142

[53] R. Browaeys , W. Saelens , Y. Saeys , Nat. Methods 2020, 17, 159. 31819264 10.1038/s41592-019-0667-5

[54] S. Jin , M. V. Plikus , Q. Nie , Nat. Protoc. 2025, 20, 180. 39289562 10.1038/s41596-024-01045-4

[55] a) C. Chen , R. Wang , X. Chen , Y. Hou , J. Jiang , Front. Oncol. 2022, 12, 924740; 35860564 10.3389/fonc.2022.924740 PMC9289165

[56] S. Tabasum , D. Thapa , A. Giobbie‐Hurder , J. L. Weirather , M. Campisi , P. J. Schol , X. Li , J. Li , C. H. Yoon , M. P. Manos , D. A. Barbie , F. S. Hodi , Cancer Immunol. Res. 2023, 11, 1493. 37728484 10.1158/2326-6066.CIR-23-0171 PMC10618652

[57] I. W. Cheuk , M. T. Siu , J. C. Ho , J. Chen , V. Y. Shin , A. Kwong , Am. J. Cancer Res. 2020, 10, 211. 32064162 PMC7017729

[58] D. Wawrzyniak , M. Grabowska , P. Głodowicz , K. Kuczyński , B. Kuczyńska , A. Fedoruk‐Wyszomirska , K. Rolle , PLoS One 2020, 15, e0237889. 32817625 10.1371/journal.pone.0237889 PMC7440653

[59] Y. Shen , B. Zhang , X. Wei , X. Guan , W. Zhang , Int. Immunopharmacol. 2022, 103, 108454. 34929481 10.1016/j.intimp.2021.108454

[60] D. Li , M. Wu , Signal Transduction Targeted Ther. 2021, 6, 291. 10.1038/s41392-021-00687-0 PMC8333067 34344870

[61] S.‐J. Liao , Y.‐H. Zhou , Y. Yuan , D. Li , F.‐H. Wu , Q. Wang , J.‐H. Zhu , B. Yan , J.‐J. Wei , G.‐M. Zhang , Breast Cancer Res. Treat. 2012, 133, 853. 22042369 10.1007/s10549-011-1844-0

[62] S. Kaur , D. D. Roberts , Semin. Cell Dev. Biol. 2024, 155, 22. 37258315 10.1016/j.semcdb.2023.05.008 PMC10684827

[63] X. Dong , Y. Yang , G. Xu , Z. Tian , Q. Yang , Y. Gong , G. Wu , Cancer Med. 2022, 11, 1371. 35037412 10.1002/cam4.4545 PMC8894706

[64] Y. Huang , V. Mohanty , M. Dede , K. Tsai , M. Daher , L. Li , K. Rezvani , K. Chen , Nat. Commun. 2023, 14, 4883. 37573313 10.1038/s41467-023-40457-w PMC10423258

[65] B. Arneth , Medicina 2019, 56, 15. 31906017 10.3390/medicina56010015 PMC7023392

[66] S. S. Badve , Y. Gökmen‐Polar , Expert Opin. Ther. Targets 2023, 27, 447. 37395003 10.1080/14728222.2023.2230362

[67] M. Chernyavska , C. K. J. C. Hermans , C. Chan , N. Baumann , T. Rösner , J. H. W. Leusen , T. Valerius , W. P. R. Verdurmen , Organs‐on‐a‐Chip 2022, 4, 100019.

[68] B. Subia , U. R. Dahiya , S. Mishra , J. Ayache , G. V. Casquillas , D. Caballero , R. L. Reis , S. C. Kundu , J. Controlled Release 2021, 331, 103. 10.1016/j.jconrel.2020.12.057 PMC8172385 33417986

[69] a) D. Truong , J. Puleo , A. Llave , G. Mouneimne , R. D. Kamm , M. Nikkhah , Sci. Rep. 2016, 6, 34094; 27678304 10.1038/srep34094 PMC5039718

[70] a) D. Truong , R. Fiorelli , E. S. Barrientos , E. L. Melendez , N. Sanai , S. Mehta , M. Nikkhah , Biomaterials 2019, 198, 63; 30098794 10.1016/j.biomaterials.2018.07.048 PMC6353712

[71] T. J. M. Manoharan , K. Ravi , A. P. Suresh , A. P. Acharya , M. Nikkhah , Adv. Healthcare Mater. 2024, 13, 2303658. 10.1002/adhm.202303658 PMC11146602 38358061

[72] A. Zhang , Y. Qian , Z. Ye , H. Chen , H. Xie , L. Zhou , Y. Shen , S. Zheng , Cancer Med. 2017, 6, 463. 28097809 10.1002/cam4.993 PMC5313646

[73] G. Gunaydin , Front. Oncol. 2021, 11, 668349. 34336660 10.3389/fonc.2021.668349 PMC8317617

[74] E. Azizi , A. J. Carr , G. Plitas , A. E. Cornish , C. Konopacki , S. Prabhakaran , J. Nainys , K. Wu , V. Kiseliovas , M. Setty , K. Choi , R. M. Fromme , P. Dao , P. T. McKenney , R. C. Wasti , K. Kadaveru , L. Mazutis , A. Y. Rudensky , D. Pe'er , Cell 2018, 174, 1293. 29961579 10.1016/j.cell.2018.05.060 PMC6348010

[75] R. Zhang , F. Qi , F. Zhao , G. Li , S. Shao , X. Zhang , L. Yuan , Y. Feng , Cell Death Dis. 2019, 10, 273. 30894509 10.1038/s41419-019-1435-2 PMC6426970

[76] E. R. Boghaert , X. Lu , P. E. Hessler , T. P. McGonigal , A. Oleksijew , M. J. Mitten , K. Foster‐Duke , J. A. Hickson , V. E. Santo , C. Brito , T. Uziel , K. S. Vaidya , Neoplasia 2017, 19, 695. 28787674 10.1016/j.neo.2017.06.004 PMC5545812

[77] A. A. Badawy , Biosci. Rep. 2022, 42, BSR20221682.36286592

[78] N. C. D'Amato , T. J. Rogers , M. A. Gordon , L. I. Greene , D. R. Cochrane , N. S. Spoelstra , T. G. Nemkov , A. D'Alessandro , K. C. Hansen , J. K. Richer , Cancer Res. 2015, 75, 4651. 26363006 10.1158/0008-5472.CAN-15-2011 PMC4631670

[79] Y. Li , M. Wang , L. Zhao , C. Liang , W. Li , Heliyon 2023, 9, e17216. 37383199 10.1016/j.heliyon.2023.e17216 PMC10293725

[80] L. Xue , C. Wang , Y. Qian , W. Zhu , L. Liu , X. Yang , S. Zhang , D. Luo , Int. Immunopharmacol. 2023, 125, 111196. 37972471 10.1016/j.intimp.2023.111196

[81] M. Al‐Mansoob , I. Gupta , R. Stefan Rusyniak , A. Ouhtit , J. Cell. Mol. Med. 2021, 25, 2309. 33486887 10.1111/jcmm.16296 PMC7933956

[82] N. N. V. Radharani , A. S. Yadav , R. Nimma , T. V. S. Kumar , A. Bulbule , V. Chanukuppa , D. Kumar , S. Patnaik , S. Rapole , G. C. Kundu , Cancer Cell Int. 2022, 22, 122. 35300689 10.1186/s12935-022-02527-9 PMC8932105

[83] V. Pelekanou , F. Villarroel‐Espindola , K. A. Schalper , L. Pusztai , D. L. Rimm , Breast Cancer Res. 2018, 20, 154. 30558648 10.1186/s13058-018-1076-x PMC6298021

[84] S. Lauvrak , E. Munthe , S. Kresse , E. Stratford , H. Namløs , L. Meza‐Zepeda , O. Myklebost , Br. J. Cancer 2013, 109, 2228. 24064976 10.1038/bjc.2013.549 PMC3798956

[85] S. Sousa , R. Brion , M. Lintunen , P. Kronqvist , J. Sandholm , J. Mönkkönen , P.‐L. Kellokumpu‐Lehtinen , S. Lauttia , O. Tynninen , H. Joensuu , D. Heymann , J. A. Määttä , Breast Cancer Res. 2015, 17, 101. 26243145 10.1186/s13058-015-0621-0 PMC4531540

[86] S.‐Q. Qiu , S. J. H. Waaijer , M. C. Zwager , E. G. E. de Vries , B. van der Vegt , C. P. Schröder , Cancer Treat. Rev. 2018, 70, 178. 30227299 10.1016/j.ctrv.2018.08.010

[87] Y. Hao , T. Stuart , M. H. Kowalski , S. Choudhary , P. Hoffman , A. Hartman , A. Srivastava , G. Molla , S. Madad , C. Fernandez‐Granda , R. Satija , Nat. Biotechnol. 2024, 42, 293. 37231261 10.1038/s41587-023-01767-y PMC10928517

[88] C. S. McGinnis , L. M. Murrow , Z. J. Gartner , Cell Syst. 2019, 8, 329. 30954475 10.1016/j.cels.2019.03.003 PMC6853612

[89] E. Becht , L. McInnes , J. Healy , C. A. Dutertre , I. W. H. Kwok , L. G. Ng , F. Ginhoux , E. W. Newell , Nat. Biotechnol. 2019, 37, 38. 10.1038/nbt.4314 30531897

[90] I. Korsunsky , N. Millard , J. Fan , K. Slowikowski , F. Zhang , K. Wei , Y. Baglaenko , M. Brenner , P. R. Loh , S. Raychaudhuri , Nat. Methods 2019, 16, 1289. 31740819 10.1038/s41592-019-0619-0 PMC6884693

[91] M. D. Robinson , D. J. McCarthy , G. K. Smyth , Bioinformatics 2010, 26, 139. 19910308 10.1093/bioinformatics/btp616 PMC2796818

[92] A. Subramanian , P. Tamayo , V. K. Mootha , S. Mukherjee , B. L. Ebert , M. A. Gillette , A. Paulovich , S. L. Pomeroy , T. R. Golub , E. S. Lander , J. P. Mesirov , Proc. Natl. Acad. Sci. USA 2005, 102, 15545. 16199517 10.1073/pnas.0506580102 PMC1239896

[93] A. Liberzon , A. Subramanian , R. Pinchback , H. Thorvaldsdóttir , P. Tamayo , J. P. Mesirov , Bioinformatics 2011, 27, 1739. 21546393 10.1093/bioinformatics/btr260 PMC3106198

[94] Z. Fang , X. Liu , G. Peltz , Bioinformatics 2022, 39, btac757.

[95] G. Yu , L. G. Wang , Y. Han , Q. Y. He , OMICS 2012, 16, 284. 22455463 10.1089/omi.2011.0118 PMC3339379

[96] A. Kassambara , M. Kosinski , P. Biecek , S. Fabian , R Package Version 0.4 2021, 9, 2021.

[97] K. Street , D. Risso , R. B. Fletcher , D. Das , J. Ngai , N. Yosef , E. Purdom , S. Dudoit , BMC Genomics 2018, 19, 477. 29914354 10.1186/s12864-018-4772-0 PMC6007078

[98] a) C. Trapnell , D. Cacchiarelli , J. Grimsby , P. Pokharel , S. Li , M. Morse , N. J. Lennon , K. J. Livak , T. S. Mikkelsen , J. L. Rinn , Nat. Biotechnol. 2014, 32, 381; 24658644 10.1038/nbt.2859 PMC4122333

