# Supplementary material for: Tumor Microenvironment On‐A‐Chip and Single‐Cell Analysis Reveal Synergistic Stromal–Immune Crosstalk on Breast Cancer Progression
Source: Adv Sci (Weinh). 2025 Mar 8;12(16):2413457. doi: 10.1002/advs.202413457 (PMC12021108; doi:10.1002/advs.202413457)
Supplement: Supplementary file 1 — Supporting Information [file ADVS-12-2413457-s008.docx]

Tumor Microenvironment On-A-Chip and Single-Cell Analysis Reveal Synergistic Stromal-Immune Crosstalk on Breast Cancer Progression

*Kalpana Ravi, Yining Zhang, Lydia Sakala, Twinkle Jina Minette Manoharan, Barbara Pockaj, Joshua LaBaer, Jin G. Park, Mehdi Nikkhah**

*Supporting Information*


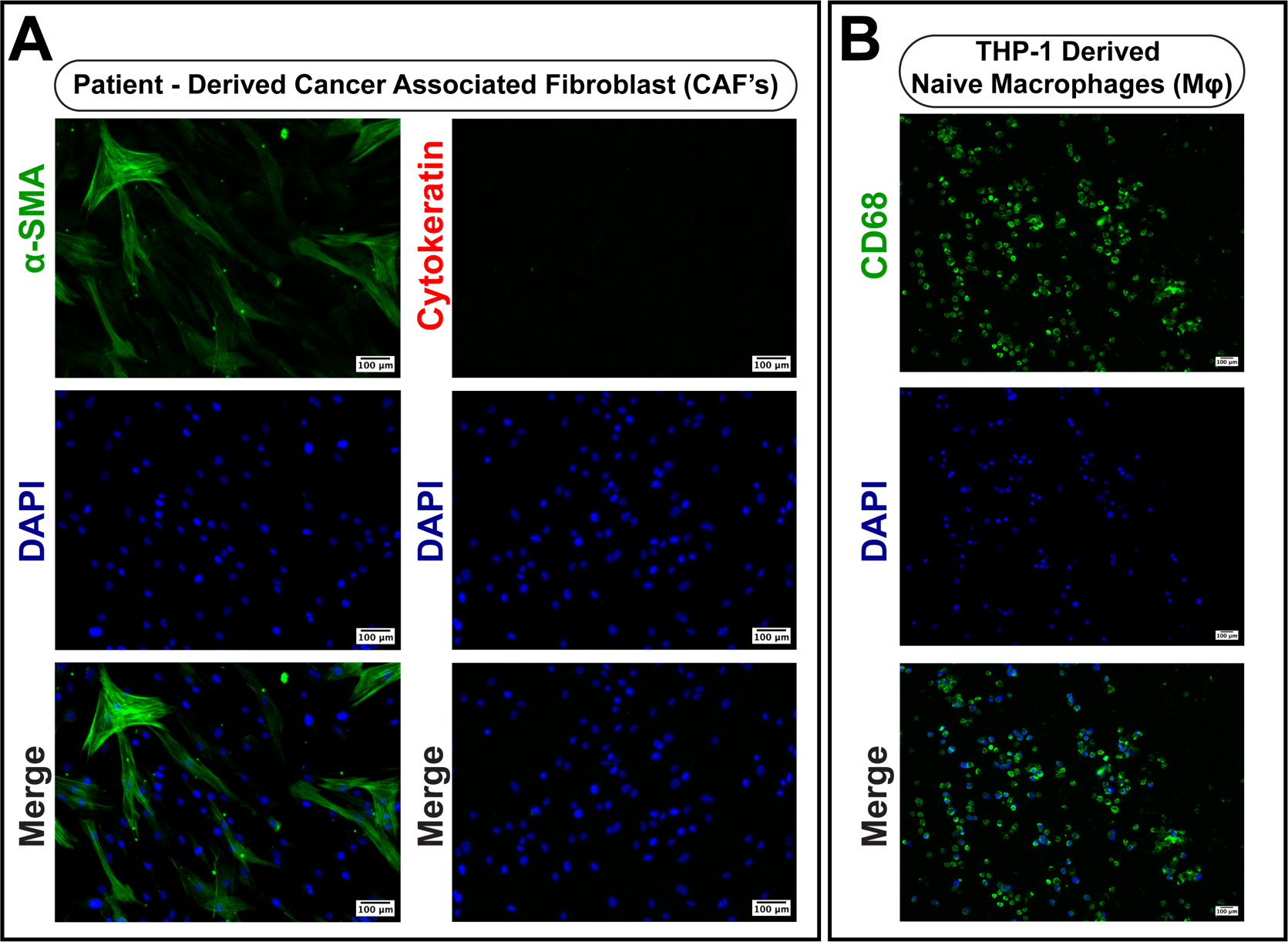
Figure S1: 2D characterization of CAFs and Mϕs. A) IF staining of patient-derived CAFs for α-SMA (green), cytokeratin (red) and counterstained with DAPI B) IF staining of THP-1 derived Mϕs for CD68 (green) counterstained with DAPI.

**
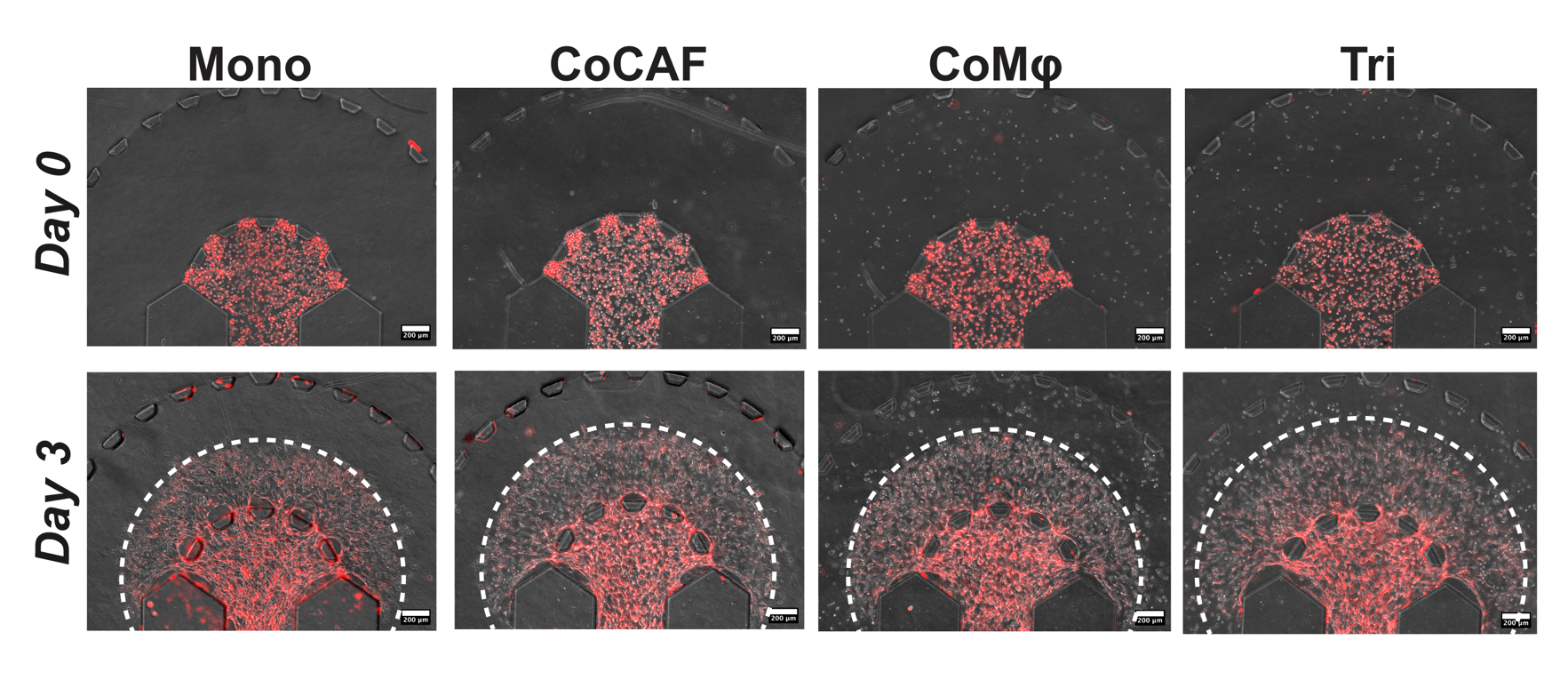
Figure S2:** Dynamic influence of CAFs and Mϕs on breast cancer invasion. Phase contrast images of Sum159 cells (red) invading the stromal region in the presence or absence of stromal cells during two different time points Day 0 (top) and Day 3 (bottom). White dashed line indicates the maximum travel distance of cancer cells.

**
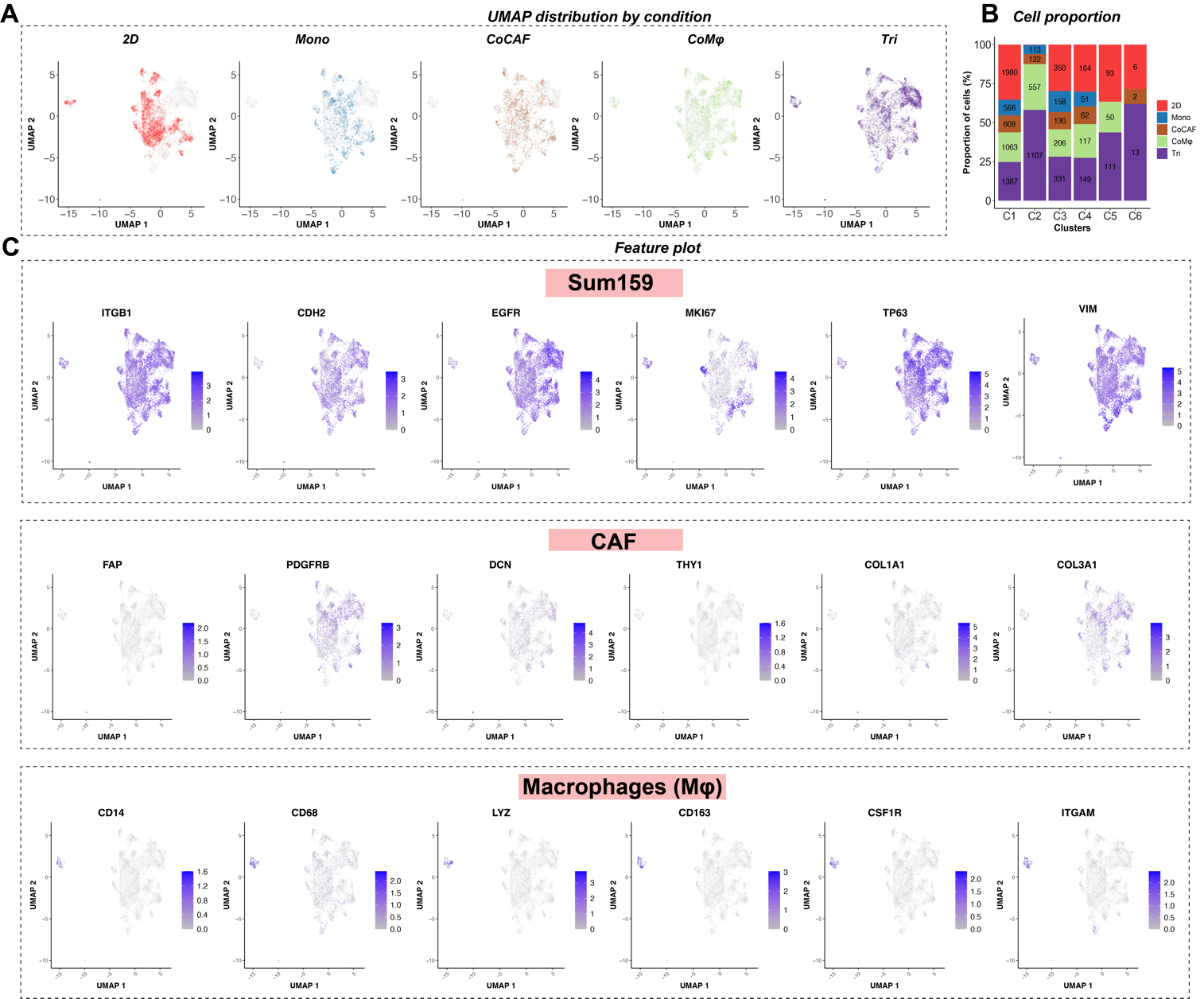
Figure S3:** scRNA-seq distribution of cells and canonical marker gene expression. A) UMAP plot of distribution of cells across different clusters based on sample type. B) Bar blot detailing the distribution of cells from each condition across clusters (2D= 2597, 3D TMEC= 6903). C) Feature plot of marker genes used for identifying Sum159 cells (top), marker genes specific to CAF (middle), and marker genes of macrophages (bottom).

**
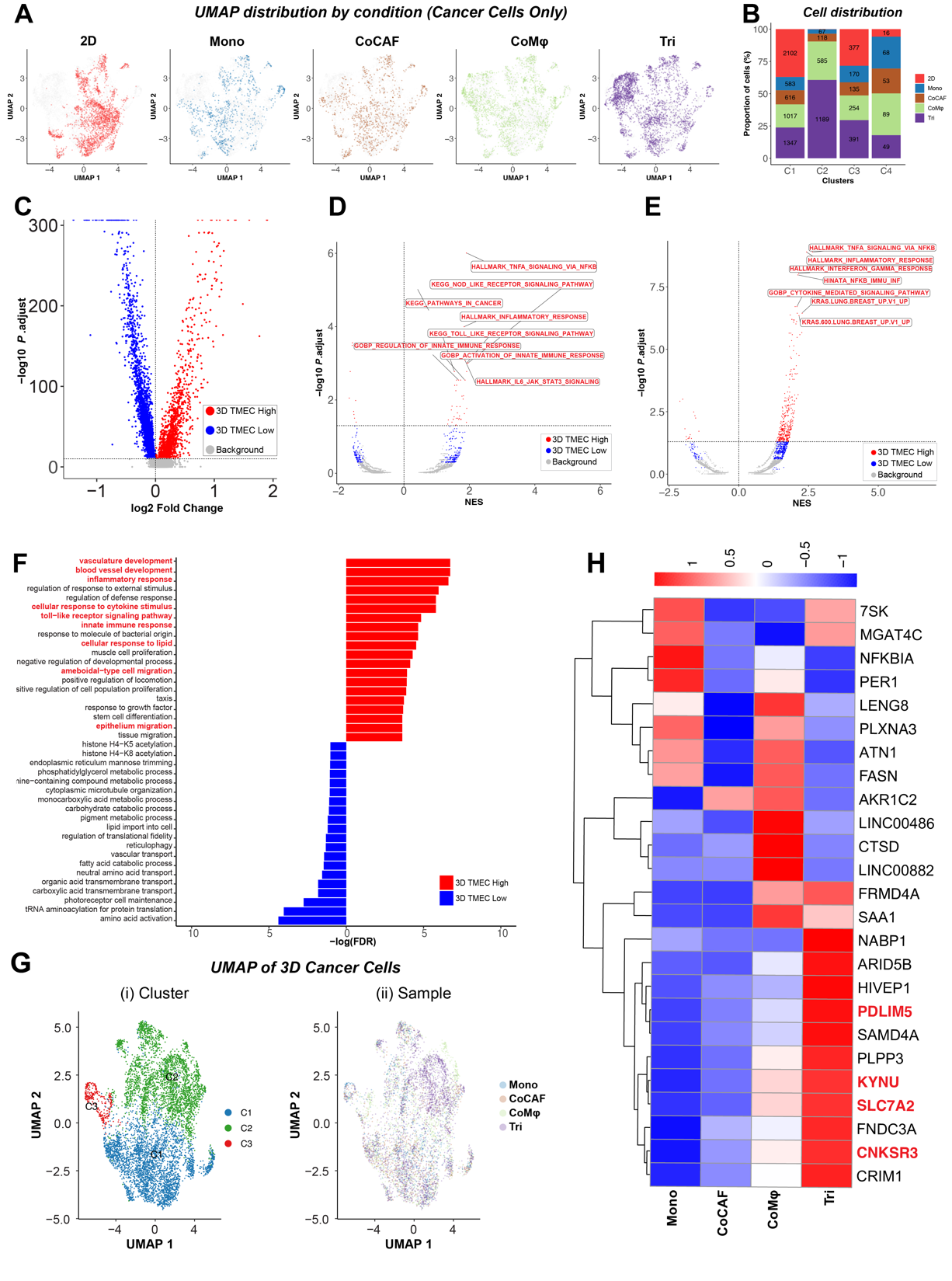
**

**Figure S4:** Transcriptomic profiling of cancer cells from 3D vs. 2D and 3D TMEC model. A) UMAP distribution of cancer cells (Sum159) across different clusters based on samples. B) Bar plot distribution of cells from each sample across all four identified clusters. C) Volcano plot of DEGs from 3D vs. 2D cancer cells (Wilcoxon test) *Red: upregulated genes in 3D (p. adjust <* 1e^-10^*), Blue: downregulated genes in 3D (p.adjust <* 1e^-10^ D) Volcano plot of enriched pathways comparing between 3D and 2D cancer cells (Wilcoxon test). *Red: p. adjust <0.05, Blue: p val <0.05. E)* Volcano plot of enriched pathways comparing between 3D and 2D cancer cells (Pseudobulk analysis). *Red: p. adjust <0.05, Blue: p val <0.05*. F) GOBP comparison of enriched terms between 3D and 2D cancer cells. Graph showing the top 20 upregulated and downregulated pathways. *Red: Upregulated DEGs, Blue: Downregulated DEGs.* G) UMAP visualization of cancer cells from the 3D model system – (i) Cluster by DEGs (ii) Cluster by sample group. H) Heatmap of top 25 DEGs identified using Wilcoxon rank-sum test. Color refers to Z-score transformed log2 expression level.

**
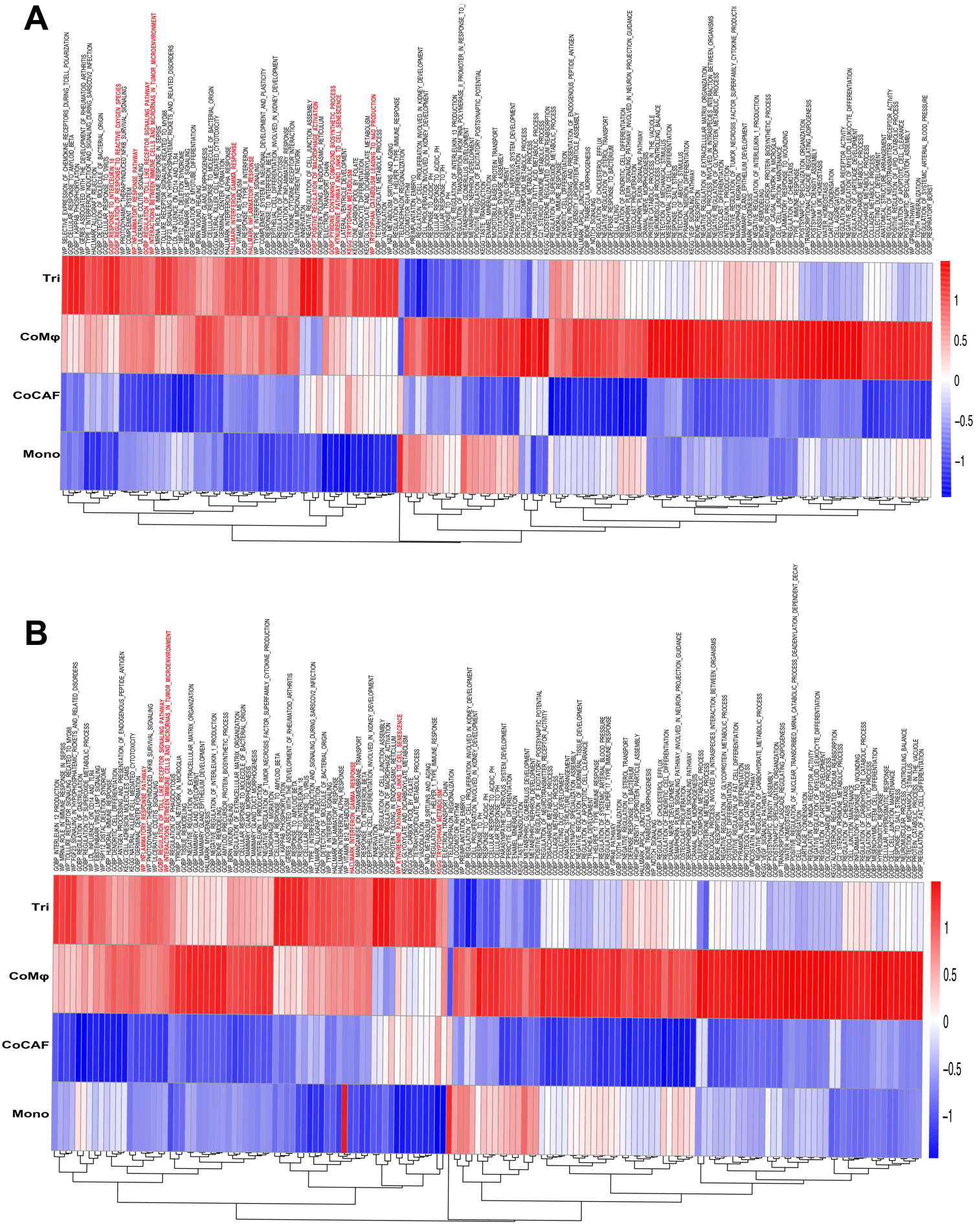
Figure S5:** ssGSEA analysis of DEGs performed on 3D cancer cells. Top 150 enriched pathways of cancer cells identified using A) t-test B) Wilcoxon test. Color refers to Z-score transformed mean of NES.

**
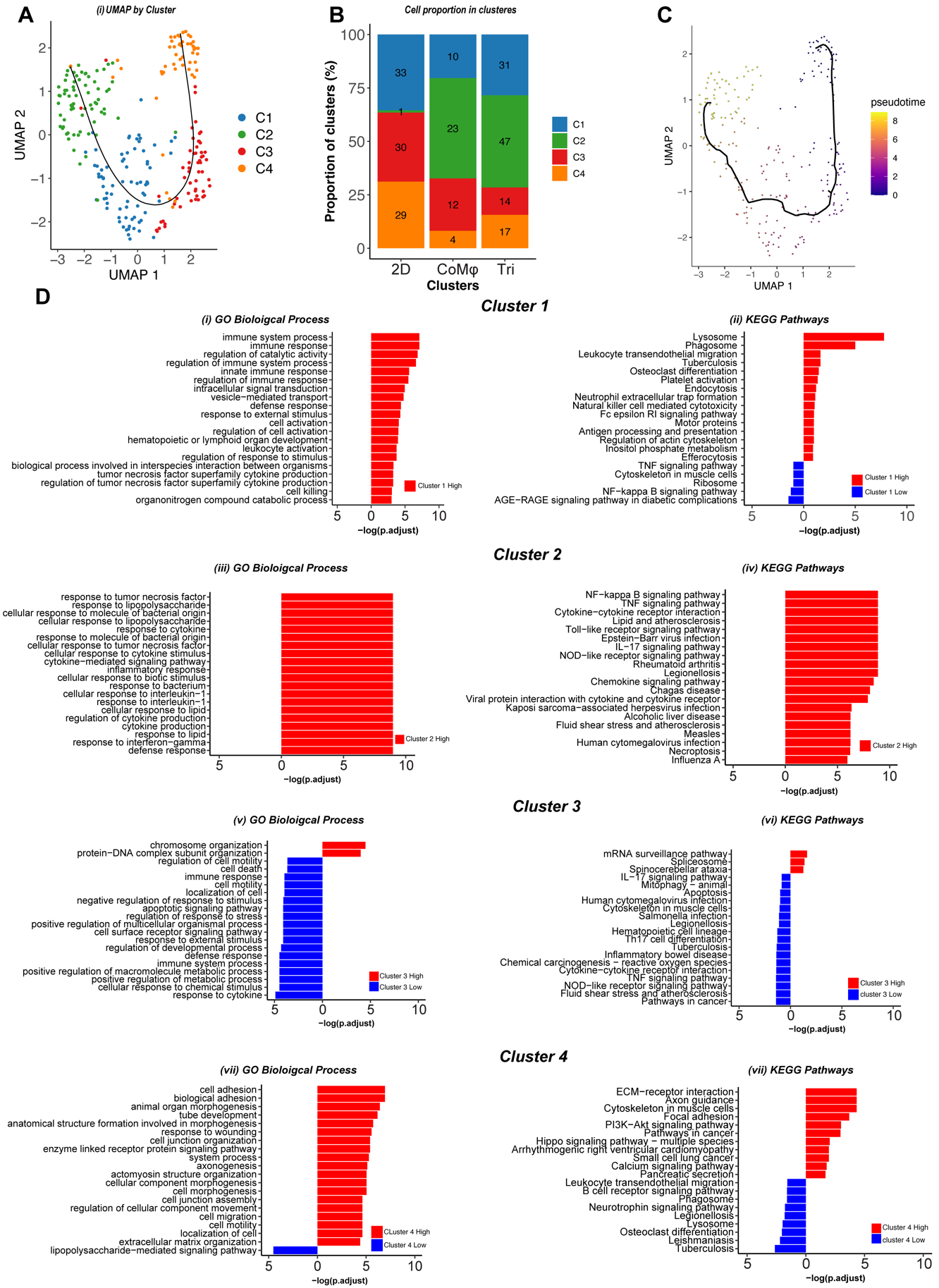
Figure S6:** Phenotype of macrophages from 3D and 2D samples. A) UMAP plot showing the clusters of Mϕs from 3D and 2D samples. B) Bar plot distribution of Mϕs in each cluster across different culture conditions. C) Pseudotime trajectory plot of Mϕs polarization with a single trajectory done by *Monocle3*. D) Barplot of enriched pathways for each cluster of Mϕs. (i-ii) Gene ontology Biological Process (left) and KEGG pathway (right) enriched in cluster 1 (C1). (iii-iv) Gene ontology Biological Process (left) and KEGG pathway (right) enriched in cluster 2 (C2). (v-vi) Gene ontology Biological Process (left) and KEGG pathway (right) enriched in cluster 3 (C3). (vii-viii) Gene ontology Biological Process (left) and KEGG pathway (right) enriched in cluster 4 (C4).

**
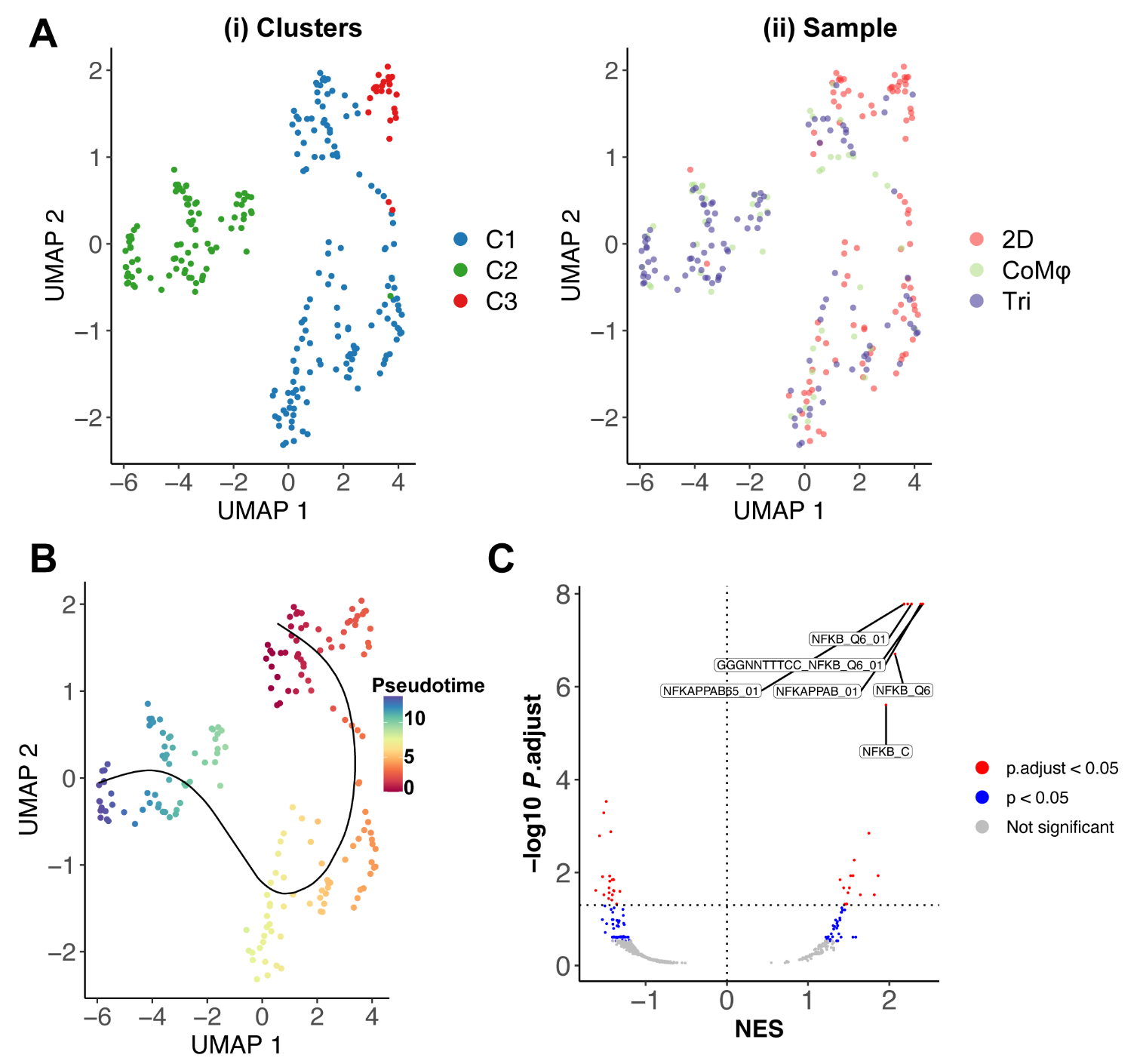
Figure S7:** Transcriptional inference on macrophage states across trajectory. A) UMAP plot of macrophages based on selected four positive and negative correlated genes by (i) clusters and (ii) sample. B) Pseudotime trajectory analysis done by Slingshot. The red dot represents the initial starting point, and the blue dot represents the end of the trajectory. C) Volcano plot of GSEA analysis showing the enrichment of transcription factor. *Red: p. adjust <0.05, Blue: p. val <0.05*.

**
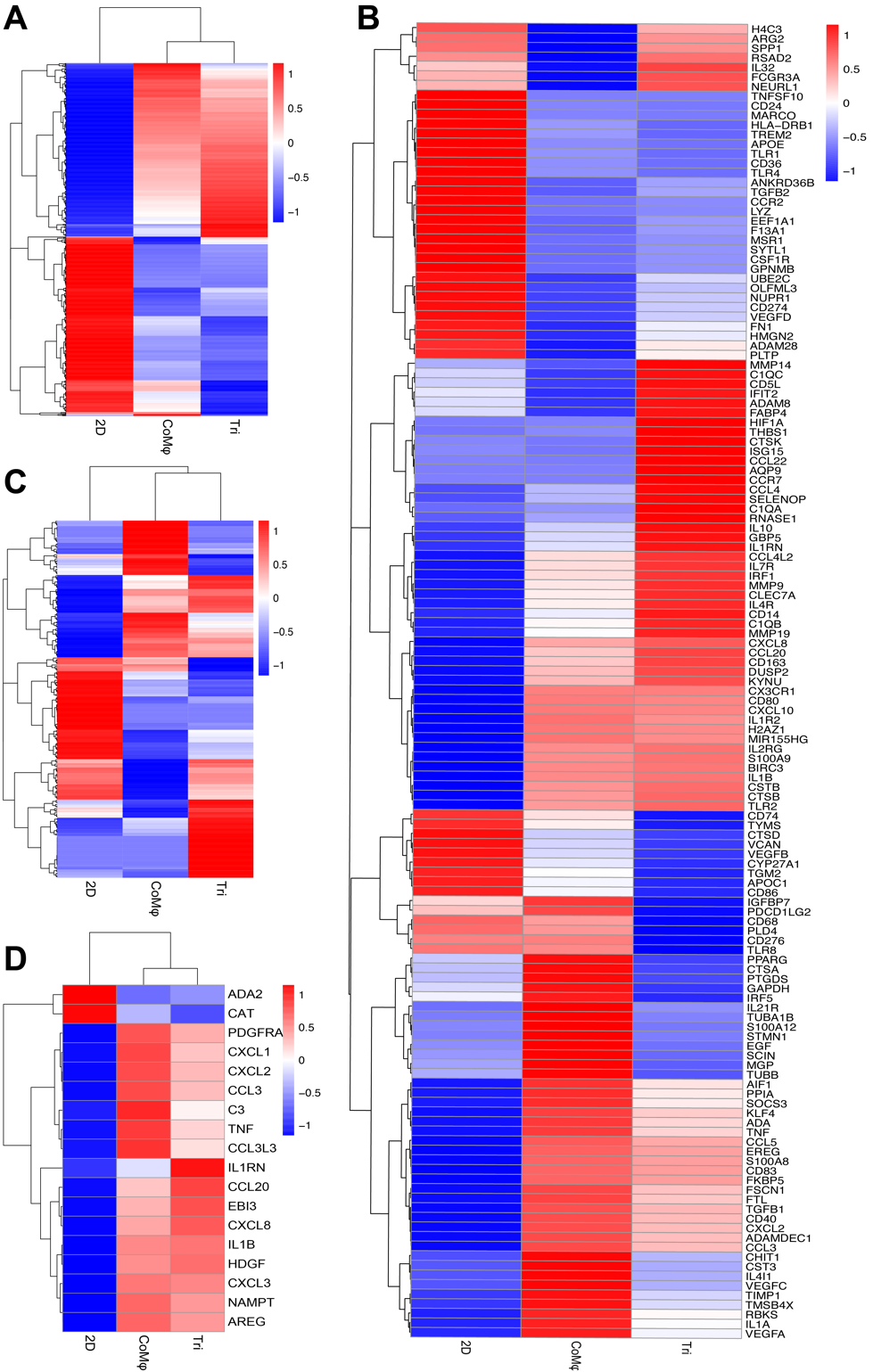
**

**Figure S8:** Differential expression analysis of Mϕs from 3D and 2D samples. A) Heatmap of DEG across different conditions. B) Heatmap of extended marker genes specific for Mϕs tumor heterogeneity. C) Heatmap of DEGs involved in Mϕs cytokine across different conditions. D) Heatmap of top 25 DEGs of cytokine across 2D, CoMϕ, and Tri. Color refers to Z-score transformed log2 expression level.


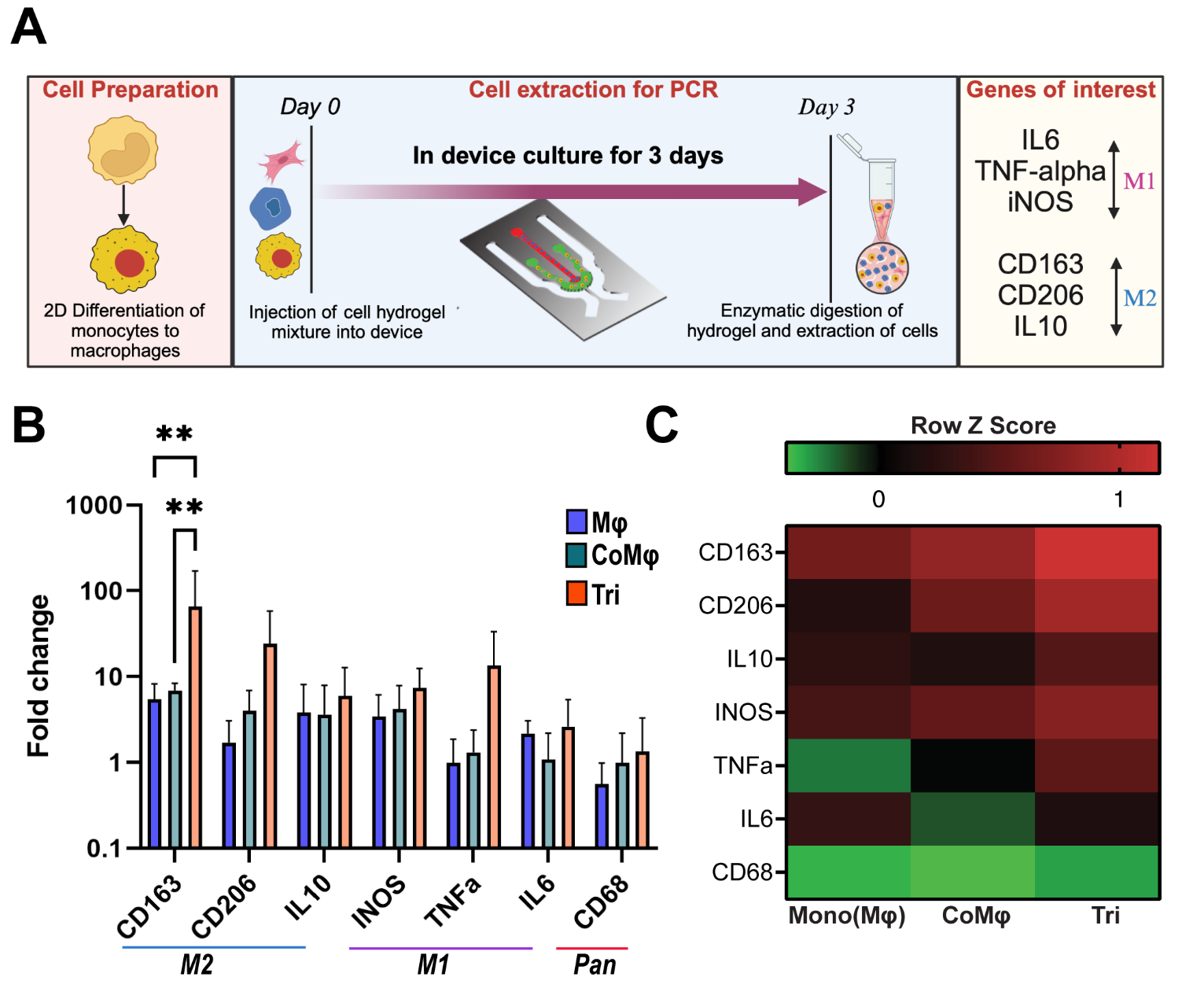
Figure S9: qPCR analysis of macrophage polarization state from Co and Tri-culture. A) Schematic representation of the experimental workflow for extraction of cells for molecular analysis with desired gene sets. Created with [BioRender.com](http://biorender.com/)  B) Quantification of foldchange of gene signatures from different culture conditions. C) Heatmap showing the relative expression of genes from Mono(Mϕ), CoMϕ, and Tri. **p< 0.01, Two-way ANOVA with Tukey’s multiple comparisons test, *n=3* biological replicates.

**
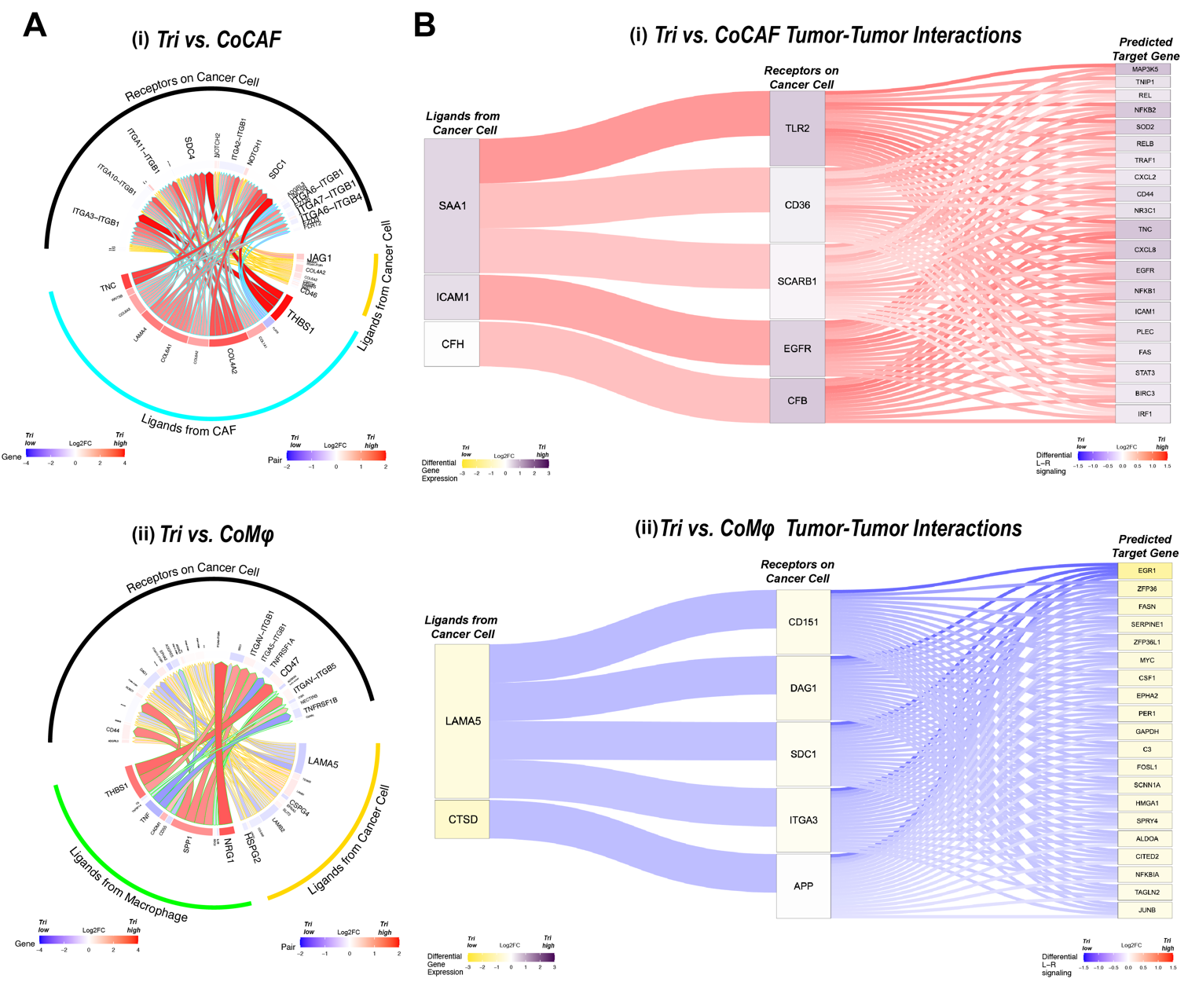
**

**Figure S10:** L-R Pair and Autocrine cell-cell interaction. A) Circos plot of L-R interaction from CellChat. (i) comparison between Tri vs. CoCAF, (ii) Comparison between Tri vs. CoMϕ. B) Prioritized L-R interaction between tumor cells and predicted target genes from Nichenet analysis. (i) Sankey plot of identified L-R interaction interactions between tumor cells and predicted target genes from Tri vs. CoCAF. (ii) Sankey plot of identified L-R interaction interactions between tumor cells and predicted target genes from Tri vs. CoMϕ.


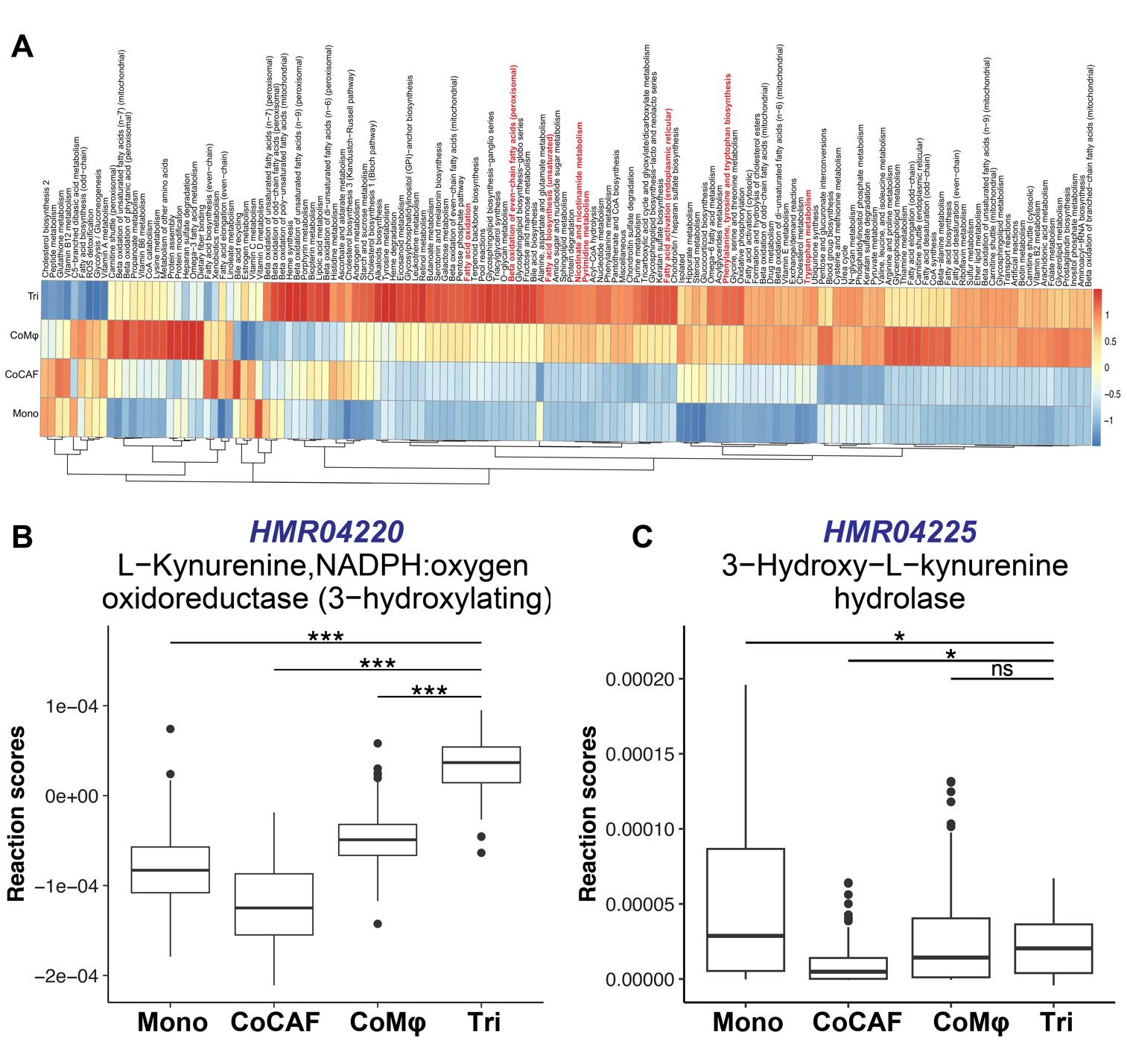


Figure S11: Metabolic Flux analysis (MFA) of cancer cells. A) MFA shows upregulation of several metabolic-related pathways. Color refers to Z-score transformed pathway activity score. B) Predicted flux scores of *KMO* reaction HMR04224 in each sample C) Predicted scores of *KYNU* reaction HMR04225 in each sample. * p. adjust < 1e^-2^, ** p. adjust <1e^-6^, *** p. adjust < 1e^-10^, ns p. adjust > 1e^-2^.


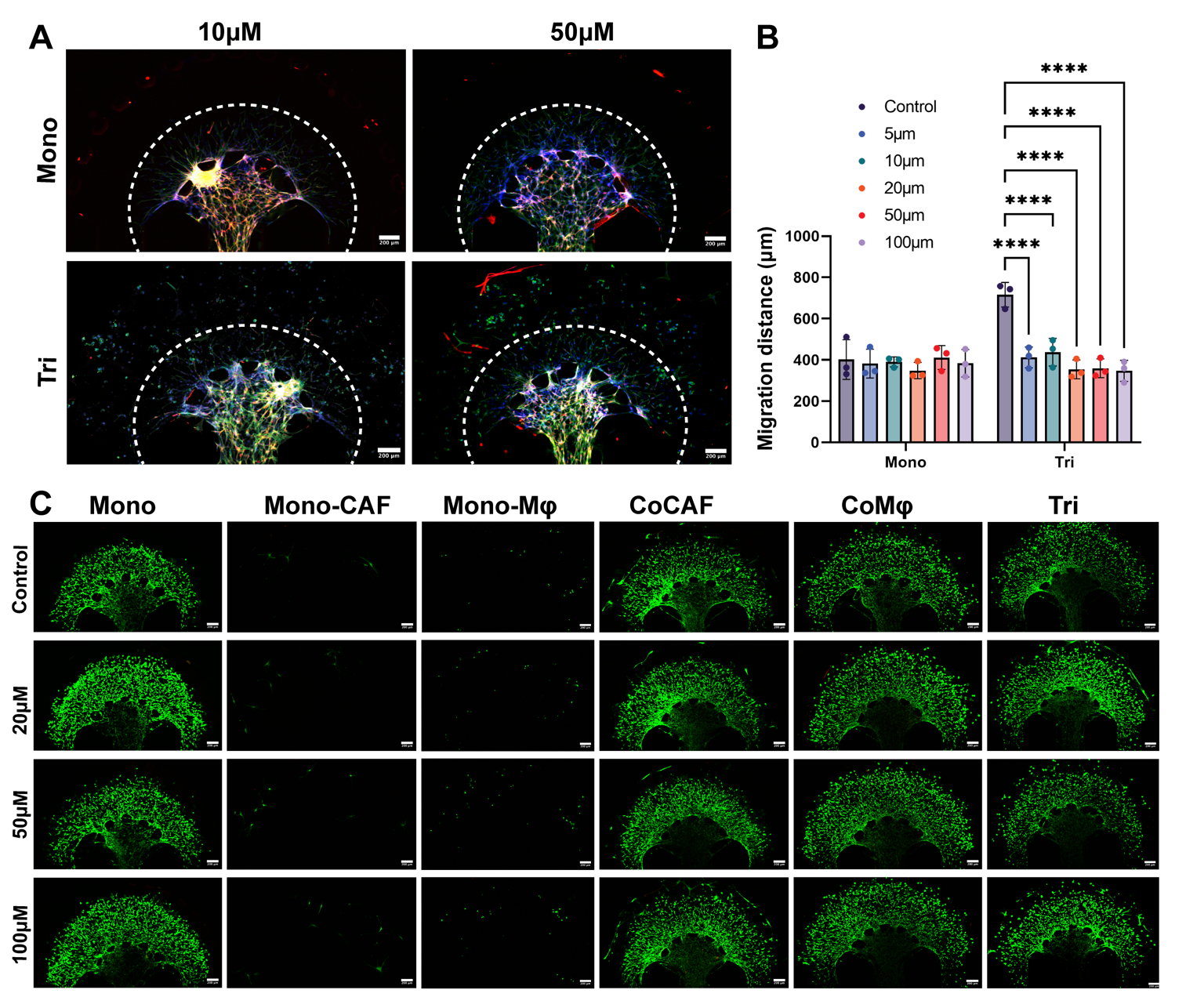
Figure S12: Pharmacological validation of KP Pathway identified through scRNA-seq. A) F-actin images of cells within the TMEC images after the addition of the drug (Ro 61-8048). Images captured on day 4. (Green- Actin, Red – Sum159, Blue – DAPI). B) Quantification of migration distance of cancer cells. Comparison of cancer cell migration within the group under various concentrations of drugs. C) Immunofluorescent viability staining of cells within the TMEC model across different experimental conditions post-drug treatment (live-green, dead-red). *n=3*

| **Experimental Conditions** | **Tumor Region** | | **Stroma Region** | |
| --- | --- | --- | --- | --- |
|  | **ECM** | **Cell type** | **ECM** | **Cell type** |
| Monoculture | Collagen:Matrigel®  (Collagen: 1mg/mL) | Sum 159  (10 million /mL) | Collagen (2mg/mL) | Acellular  (No cells) |
| Co-culture w/ CAF  (CoCAF) | Collagen:Matrigel®  (Collagen: 1mg/mL) | Sum 159  (10 million /mL) | Collagen (2mg/mL) | CAFs  (25K cells/mL) |
| Co-culture w/ Mϕ  (CoMϕ) | Collagen:Matrigel®  (Collagen: 1mg/mL) | Sum 159  (10 million /mL) | Collagen (2mg/mL) | Mϕ  (0.5 million/mL) |
| Tri-culture | Collagen:Matrigel®  (Collagen: 1mg/mL) | Sum 159  (10 million /mL) | Collagen (2mg/mL) | CAFs+Mϕ  (25K cells/mL + 0.5 million/mL) |

Table S1: Experimental conditions for invasion assay, proliferation, viability, live imaging, and sc-RNA sequencing

Table S2: DEG_t-test

Table S3: DEG_wilcoxon test

Table S4: GSEA_t-test

Table S5: GSEA_wilcoxon test

Table S6: GSEA_Pseudo bulk

Table S7: GOBP Pathways_3D vs. 2D Cancer cells

Table S8: DEG_ 3D Cancer cells (*t-test* and Wilcoxon)

Table S9: ssGSEA Analysis_3D Cancer cells

Table S10: GOBP Pathways_3D Cancer cells

Table S11: GOMF Pathways_3D Cancer cells

| **M0** | **M1** | **M2** |
| --- | --- | --- |
| SELENOP | IRF1 | MMP9 |
| C1QB | KYNU | IL4R |
| AIF1 | IL1B | CLEC7A |
| C1QA | CD40 | IL1RN |
| CD36 | CCL5 | MMP19 |
| LYZ | TNF | CTSB |
| CD68 | IL1A | VEGFA |
|  | CXCL10 | CD163 |
|  | IL6 | IL10 |
|  | IRF5 | TGFB3 |
|  | CD86 | VEGFC |
|  |  | IL1R2 |
|  |  | CCL4 |
|  |  | CCL20 |
|  |  | TGFB1 |
|  |  | PDCD1LG2 |
|  |  | CTSA |
|  |  | EGF |
|  |  | LYVE1 |
|  |  | CSF1R |
|  |  | CD276 |
|  |  | CD274 |
|  |  | MSR1 |
|  |  | VEGFB |
|  |  | CTSD |
|  |  | ARG2 |
|  |  | VEGFD |
|  |  | FN1 |
|  |  | TGFB2 |
|  |  | TNFSF12 |
|  |  | MMP14 |
|  |  | WNT7B |

Table S12: M0, M1, and M2 genes signature used for scRNA analysis

| Gene | Reference |
| --- | --- |
| *FCN1, S100A8, S100A9, CD300E, CD14, FCGR3A, CD74, HLA-DRA, HLA-DPB1, CD68, APOE, C1QA, C1QB, C1QC, SPP1, TREM2, CD163, IL1B, CXCL8, CXCL2, CXL16, IL7R, IL2RG, IL32, IL4L1, CXCL10, CXCL9, CXCL11, PLA2G2D, MMP9, CCL2, CX3CR1, CXCL12, UBD, PTGDS, IGLC2, CCL2, FN1, TNFSF10, MARCO,TNF, CCL8, UBE2C, TYMS, STMN1, TUBB, HMGN2, PPIA, GAPDH, ANKRD36B, MTRNR2L12, F13A1,HLA-DPA1, FDCSP, ADAM8, MGP, PLTP, RNASE1, IGFBP7, THBS1, EREG, NEURL1, CEACAM3, LYPD2, CD79B, SYTL1, EEF1A1, MSLN, CLDN4, SYNDIG1, MCF2L, CD24, CH25H, SCIN, FKBP5, SPP1, CCL4L2, CCL3L1, CL4, ADAMDEC1, MIR155HG, OLFML3, RBKS, DUSP2, IL1B, CCL3, CD83, CETP, LYVE1, CD5L, CXCL12, MRC1, FOLR2, SLC40A1, SELENOP, ADAM28, PLD4, NUPR1, GPNMB, CTSD, S100A12, VCAN, CHIT1, PTGDS IL2RG, CCL18, UBD, CYP27A1, MMP9, CSTB, CCL7, APOBEC3A, AQP9, RSAD2, TIMP1, TMSB4X, FTL, IL7R, IL2RG, IL32, IL4I1, CXCL10, CXCL9, CXCL11, PLA2G2D, MMP9, CCL2, CX3CR1, CXCL12, UBD, PTGDS, IGLC2, APOE, MARCO, TNFSF10, FN1, TNF, CCL8, CCL5, GBP5* | ^[1]^ |
| *TUBA1B, C1QC, C1QB, IL1B, CCL3, CXCL11, CXCL10, VCAN, S100A8, SLC40A1, FOLR2, APOC1, ACP5* | ^[2]^ |
| *CD68, CD14, FCGR3A, CST3, HLA-DRA, FCER1A,S100A8, S100A9, EREG, FTL, APOC1, APOE, SELENOP, FLOR2, C1QB, SPP1, NUPR1, CSTB, IL32, CD3D, CD7, CXCL10, IFIT2, ISG15, STMN1, H2AFZ, HIST1H4C, CCR7, FSCN1, BIRC3, CTSK, MMP9, ACP5, IGHG1, IGKC* | ^[3]^ |

Table S13: Expanded Mϕ marker genes list

Table S14: Enriched Pathways_Comparison between clusters

Table S15: GSEA_Correlated genes for trajectory

| **Experimental Conditions** | **Tumor Region** | | **Stroma Region** | |
| --- | --- | --- | --- | --- |
|  | **ECM** | **Cell type** | **ECM** | **Cell type** |
| Monoculture  (Mϕ) | Collagen:Matrigel®  (Collagen: 1mg/mL) | Acellular  (No cells) | Collagen (2mg/mL) | Mϕ  (0.5 million/mL) |
| Co-culture w/ CAF  (CoMϕ) | Collagen:Matrigel®  (Collagen: 1mg/mL) | Sum 159  (10 million /mL) | Collagen (2mg/mL) | Mϕ  (0.5 million/mL) |
| Tri-culture | Collagen:Matrigel®  (Collagen: 1mg/mL) | Sum 159  (10 million /mL) | Collagen (2mg/mL) | CAFs+Mϕ  (25K cells/mL + 0.5 million/mL) |

Table S16: Experimental conditions for qRT-PCR

| **Genes (Human)** | **Forward sequence (5’ – 3’)** | **Reverse sequence (3’ - 5’)** |
| --- | --- | --- |
| GAPDH | ACCCAGAAGACTGTGGATGG | CAGTGAGCTTCCCGTTCAG |
| CD68 | CTTCTCTCATTCCCCTATGGACA | GAAGGACACATTGTACTCCACC |
| iNOS | TCCAAGGTATCCTGGAGCGA | CAGGGACGGGAACTCCTCTA |
| TNFa | AGGACCAGCTAAGAGGGAGA | CCCGGATCATGCTTTCAGTG |
| IL6 | ACTCACCTCTTCAGAACGAATTG | GTCGAGGATGTACCGAATTTGT |
| CD163 | TTTGTCAACTTGAGTCCCTTCAC | TCCCGCTACACTTGTTTTCAC |
| CD206 | GGGTTGCTATCACTCTCTATGC | TTTCTTGTCTGTTGCCGTAGTT |
| IL10 | GACTTTAAGGGTTACCTGGGTTG | TCACATGCGCCTTGATGTCTG |

Table S17: Primers sequence used for qRT-PCR

Table S18: KEGG Pathways_Macrophage

Table S19: GOBP Pathways_Macrophages

Movie S1.

Time-lapse imaging of Sum159 cells (red) migrating through the 3D stroma within the TMEC model in the absence of CAFs and Mϕ (Mono condition), captured for a total duration of 16h with an interval of 45 minutes.

Movie S2.

Time-lapse imaging of Sum159 (red) cells migrating through the 3D stroma within the TMEC model in the presence of CAFs (unlabeled) in CoCAF condition, captured for a total duration of 16h with an interval of 45 minutes.

Movie S3.

Time-lapse imaging of CAFs (unlabeled) migrating through the 3D stroma within the TMEC model in the presence of Sum159 cells (CoCAF condition), captured for a total duration of 16h with an interval of 45 minutes.

Movie S4.

Time-lapse imaging of Sum159 cells (red) migrating through the 3D stroma within the TMEC model in the presence of Mϕ in CoMϕ condition, captured for a total duration of 16h with an interval of 45 minutes.

Movie S5.

Time-lapse imaging of Mϕs migrating through the 3D stroma within the TMEC model in the presence of Sum159 cells (CoMϕ condition), captured for a total duration of 16h with an interval of 45 minutes.

Movie S6.

Time-lapse imaging of Sum159 cells (red) migrating through the 3D stroma within the TMEC model in the presence of CAFs and Mϕs in Tri condition, captured for a total duration of 16h with an interval of 45 minutes.

Movie S7.

Time-lapse imaging of CAFs migrating through the 3D stroma within the TMEC model in the presence of Sum159 cells (red) and Mϕs (unlabeled) in Tri condition, captured for a total duration of 16h with an interval of 45 minutes.

Movie S8.

Time-lapse imaging of Mϕs migrating through the 3D stroma within the TMEC model in the presence of Sum159 cells (red) and CAFs (unlabeled) in Tri condition, captured for a total duration of 16h with an interval of 45 minutes.

**References:**

[1] Y. Zhang, H. Chen, H. Mo, X. Hu, R. Gao, Y. Zhao, B. Liu, L. Niu, X. Sun, X. Yu, *Cancer cell* **2021**, 39, 1578.

[2] Y. Zhang, F. Zhong, L. Liu, *Breast Cancer Research* **2024**, 26, 129.

[3] S. M. Cha, J.-W. Park, Y. J. Lee, H. J. Lee, H. Lee, I. W. Lee, G. Gong, S. H. Park, H. J. Lee, B.-K. Jeong, *NPJ Breast Cancer* **2024**, 10, 83.
